# Exploring of the feature space of *de novo* developed post-transcriptional riboregulators

**DOI:** 10.1371/journal.pcbi.1006170

**Published:** 2018-08-17

**Authors:** Gert Peters, Jo Maertens, Jeroen Lammertyn, Marjan De Mey

**Affiliations:** 1 Centre for Synthetic Biology, Ghent University, Ghent, Belgium; 2 BIOSYST-MeBioS, KU Leuven, Leuven, Belgium; University of Missouri, UNITED STATES

## Abstract

Metabolic engineering increasingly depends upon RNA technology to customly rewire the metabolism to maximize production. To this end, pure riboregulators allow dynamic gene repression without the need of a potentially burdensome coexpressed protein like typical Hfq binding small RNAs and clustered regularly interspaced short palindromic repeats technology. Despite this clear advantage, no clear general design principles are available to *de novo* develop repressing riboregulators, limiting the availability and the reliable development of these type of riboregulators. Here, to overcome this lack of knowledge on the functionality of repressing riboregulators, translation inhibiting RNAs are developed from scratch. These *de novo* developed riboregulators explore features related to thermodynamical and structural factors previously attributed to translation initiation modulation. In total, 12 structural and thermodynamic features were defined of which six features were retained after removing correlations from an *in silico* generated riboregulator library. From this translation inhibiting RNA library, 18 riboregulators were selected using a experimental design and subsequently constructed and co-expressed with two target untranslated regions to link the translation inhibiting RNA features to functionality. The pure riboregulators in the design of experiments showed repression down to 6% of the original protein expression levels, which could only be partially explained by a ordinary least squares regression model. To allow reliable forward engineering, a partial least squares regression model was constructed and validated to link the properties of translation inhibiting RNA riboregulators to gene repression. In this model both structural and thermodynamic features were important for efficient gene repression by pure riboregulators. This approach enables a more reliable *de novo* forward engineering of effective pure riboregulators, which further expands the RNA toolbox for gene expression modulation.

## Author summary

To allow reliable forward engineering of microbial cell factories, various metabolic engineering efforts rely on RNA-based technology. As such, programmable riboregulators allow dynamic control over gene expression. However, no clear design principles exist for *de novo* developed repressing riboregulators, which limits their applicability. Here, various engineering principles are identified and computationally explored. Subsequently, various design criteria are used in an experimental design, which were explored in an *in vivo* study. This resulted in a regression model that enables a more reliable computational design of repression small RNAs.

## Introduction

Over the last decade, synthetic biology and systems biology spurred major advances in metabolic engineering, resulting in several economically competitive production processes for both bulk and fine chemicals from renewable resources, revolutionizing industrial biotechnology [1, 2, 3, 4, 5]. In this context, interfering with the native metabolism of the production host is a necessity to redirect the metabolic flux towards the product of interest with a view to maximizing productivity [6, 7]. Traditionally, tuning the cellular metabolism has been done through gene deletions, which is impossible for numerous essential genes often related to various biosynthetic pathways [8, 9]. As such, maximizing various production pathways requires tools able to specifically reduce gene expression. To this end, zinc fingers and transcription activator-like effectors were engineered to dynamically control transcription of a specific gene through DNA-binding proteins [10, 11]. These custom gene expression regulators are outperformed by recently emerged clustered regularly interspaced short palindromic repeats interference (CRISPRi), an adaptation of the type II clustered regularly interspaced short palindromic repeats (CRISPR) system controlling transcription through reversible binding of a RNA-guided deactivated Cas9 nuclease to DNA [12]. Various metabolic engineering efforts in multiple organisms used this CRISPRi technology to successfully repress a series of specific genes in a dynamic way, hereby ameliorating the desired product formation [13, 14, 15].

Alternative approaches to control gene expression on the translational level employ small RNAs (sRNAs) to repress protein production by blocking translational initiation, enabling metabolic flux redirection at will [9, 16, 17, 18]. Similar to the CRISPRi technology, which requires a small guide RNA (sgRNA) able to bind to the dCas9 protein, these types of sRNAs also require a protein binding RNA motif as they rely on the stabilizing Hfq protein. This Hfq based riboregulation has been used in various metabolic engineering efforts for gene repression and uses known Hfq binding motifs to combine with an antisense part, targeting specific mRNA. The dependence on coexpressed proteins might cause increased metabolic burden, which can lead to long term genetic instability and unexpected behaviour [14, 19, 20]. To reduce these undesired effects, gene expression modulation systems are preferred that solely rely on RNA [21]. These pure riboregulators require less cellular resources by avoiding the extra translation step, hereby lowering metabolic burden [21]. For example, riboregulator technology is successfully used to precisely downregulate specific genes, hereby redirecting metabolite fluxes towards the phenotype of interest [22, 23, 24]. Also, pure riboregulators, which do not require proteins, harness large potential for the construction of fast responding RNA circuitery [17, 25, 26, 27]. However, practical applications of pure riboregulators are the result of thorough laborious, inefficient and impractical screening of multiple designs comprising hundreds of nucleotides [22, 23].

Early pure riboregulators were designed to hybridize the translation initiation region. The RNA architecture in this region plays a pivotal role in the translation initiation process, enabling gene expression through RNA-RNA interactions [28]. This apparent link between RNA structure and biological function, in combination with the ease and reliability of RNA secondary structure prediction, drove several gene silencing efforts solely using RNA. However, successful attempts to modulate gene expression using solely *trans* expressed RNA employed a variety of features [29, 30, 31]. As such, interfering with translation initiation using solely RNA-RNA interactions has been attributed to various features of the *trans* expressed RNA molecule [26, 29, 30, 32, 33, 34, 35].

These features are classified as either structural or thermodynamic features. Several structural features of riboregulators modulating translation initiation through RNA-RNA interactions include post-transcriptional ribosome binding site (RBS) occlusion [33, 34], formation of paired termini structures [32], and manipulation of the structural accessibility of the target site [29, 35]. Besides structural constraints, various thermodynamic features were previously used to design and optimize translation interfering riboregulators, mainly comprising formation and activation energies [26, 33]. Formation energies are typically obtained by estimating the minimum free energy (MFE) [33]. Despite the importance of activation energy, various estimation methods for the activation energy were previously used to create functional riboregulators [26, 33]. These methods rely on the initial monomeric structures and are based on the assumption that the unbound nucleotides in this state initiates the RNA-RNA complex formation [26, 33].

This broad range of employed features indicates the lack of consensus in literature, which limits the general applicability of the current design rules for pure riboregulators (without using coexpressed proteins). For instance, simply expressing the antisense strand does not fully repress gene expression on the post-transcriptional level [22, 23]. As such, various types of riboregulators suitable for metabolic engineering purposes were created using a number of different riboregulator design features, once again indicating the lack of consensus in literature on the development of riboregulators [26, 33, 36]. Overall, these riboregulators are either developed from a natural existing RNA regulator chassis or created *de novo*, the latter being the most interesting as this enables forward engineering in a broader context [33, 36]. Moreover, only activating riboregulators were created *de novo*, which limits the construction of genetic circuitery using solely RNA.

Here, we propose a framework for the *de novo* design of pure riboregulators, referred to as translation inhibiting RNAs (tiRNAs), which repress gene expression by blocking translation initiation. To develop this predictive framework, the influence of all features previously attributed to post-transcriptional gene modulation were analyzed in a design of experiments (DOE). This experimental design allows exploration of the feature landscape and evaluation of their influence on gene repression. First, using a library of tiRNA, all features were analyzed *in silico* to create a collection of features with maximal information content. Next, the performance of *de novo* designed tiRNAs was evaluated *in vivo*, and used to construct an ordinary least squares (OLS) and a partial least squares (PLS) model which links riboregulator features to gene repression.

## Methods

### Strains and growth conditions

*Escherichia coli* strain DH10B (Invitrogen) was used for both plasmid construction and fluorescence measurement purposes. Unless otherwise stated, all products were purchased from Sigma-Aldrich (Diegem, Belgium). For plasmid construction and fluorescence measurements strains were grown in grown in lysogeny broth (LB) and MOPS EZ rich medium (Teknova, Bioquote, York, United Kingdom) at pH 7.4, respectively at 37°C with shaking. LB was composed of 1% tryptone-peptone (Difco, Erembodegem, Belgium), 0.5% yeast extract (Difco) and 1% sodium chloride (VWR, Leuven, Belgium). LB agar (LBA) plates contain the same components as LB with the addition of 1% agar. If required, medium was supplemented with 100 *μ*g ml^-1^ ampicillin and 50 *μ*g ml^-1^ kanamycin.

### Plasmids

pSilence plasmids were medium-copy vectors (pBR322 origin of replication (ori) and ampicillin resistance marker, originating from pSB6A1 [37]) with a truncated version (all nucleotides after the transcription start were removed) of the proD [38] as promoter and BBa_B1006 [39] as terminator for tiRNA expression (see Figure A in S1 Text for more details), and pTarget plasmids were low-copy vectors (pSC101 ori and kanamycin resistance marker, originating from pCL1920 [40]) with proB [38] as promoter, *mKate2* [41] as reporter gene, rnpB T1 [39] as terminator, and the target 5’ untranslated region (UTR) (see Figure B in S1 Text for more details). The reporter mKate2 was used due to its low background and good fluorescent protein properties (brightness and maturation time) [41]. A schematic overview of the two plasmid types used in this study (pSilence and pTarget) is shown in Figure C in S1 Text.

The control plasmids used in this study were pBlank_1_ and pBlank_2_, which are the same vectors as the pSilence and pTarget plasmids, respectively. The pBlank_1_ plasmid comprises only the vector backbone and pBlank_2_ contains the *mKate2* open reading frame (ORF) and rnpB T1 [39] as terminator, thus without promoter and UTRs. All plasmids used in this study were constructed using Golden Gate [42] and CPEC [43] assembly. DNA oligonucleotides were commercially ordered from IDT (Leuven, Belgium) and DNA sequences of every constructed plasmid were verified using sequencing services (Macrogen Inc., Amsterdam, The Netherlands). All tiRNA sequences used in this study are listed in Table A in S1 Text. Details of the plasmids and DNA sequences used in this study are listed in Table B and C in S1 Text, respectively.

### *In vivo* fluorescence and optical density (OD) measurements

For *in vivo* assessment of translational inhibition, strains were plated on LBA plates containing 100 *μ*g ml^-1^ ampicillin and 50 *μ*g ml^-1^ kanamycin. After overnight incubation, three colonies were inoculated in 150 *μ*l MOPS EZ rich medium, covered by a Breathe-Easy sealing membrane (Sigma-Aldrich), and grown overnight on a Compact Digital Microplate Shaker (Thermo Scientific) at 800 rpm and 37°C. Subsequently, these cultures were 1:100 diluted in 150 *μ*l of fresh MOPS EZ rich medium and grown on a Compact Digital Microplate Shaker until late log phase (6 h) at 800 rpm and 37°C. Subsequently, fluorescence and OD were measured using a Tecan M200 pro microplate reader. Precultures were grown in Greiner bio-one (Vilvoorde, Belgium) polystyrene F-bottom 96 well plates. Fluorescence and OD measurements were performed after growth in Greiner bio-one (Vilvoorde, Belgium) black *μ*clear 96 well plates. For measuring mKate2 expression an excitation wavelength and an emission wavelength of 588 nm and 633 nm were used, respectively. OD was measured at a wavelength of 700 nm to reduce bias in estimates of cell abundance [44].

### Fluorescence data analysis

For fluorescence measurements, two types of controls were used on every 96-well microtiter plate, i.e., a MOPS EZ rich medium blank and *E. coli* DH10B cells without fluorescent protein expression (contains pBlank_1_ and pBlank_2_ plasmids). The medium blank was used to correct the background OD (OD_bg_) of the medium. The fluorescence of the strain without fluorescent protein expression (FP_bg_) was used to correct for the background fluorescence of *E. coli*. For all strains fluorescence per OD was calculated as follows:

$$(\frac{\text{FP}}{\text{OD}}{)}_{\text{corrected}}=\frac{\text{FP}-{\text{FP}}_{\text{bg}}}{\text{OD}-{\text{OD}}_{\text{bg}}}$$ (1)

The relative protein expression was defined as follows:

$$\text{Relative}\;\text{protein}\;\text{expression}\;\left(\%\right)=\frac{(\frac{\text{FP}}{\text{OD}}{)}_{\text{corrected}}\;\text{with}\;\text{riboregulator}}{(\frac{\text{FP}}{\text{OD}}{)}_{\text{corrected}}\;\text{without}\;\text{riboregulator}}\times 100$$ (2)

### Feature quantification using RNA bioinformatics

For each tiRNA candidate 12 features were calculated (see Table 1 for detailed definitions), which were determined based on various previous riboregulator efforts described in literature [26, 29, 33, 34, 35]. The tiRNA features are classified in two main groups: thermodynamic properties and structural constraints. All intra- and intermolecular interactions between RNA molecules were predicted using RNAfold [45] and RNAcofold [46], respectively. Both RNA secondary structure prediction algorithms were available through the Vienna RNA package [47] and were used with only the options –noLP -d2 and an accuracy of 10^-100^, all other settings are set to the default setting. Suboptimal structures of RNA molecules are drawn with probabilities equal to their Boltzmann weights using RNAsubopt [48]. The intermolecular binding between the unbound part of the tiRNA and the UTR is estimated by the RNAup algorithm [49]. All calculations were done using Python scripting on an Intel Xeon E5-2670 (2.60GHz) Linux (Debian) server. Details on the quantification of thermodynamic and structural properties of tiRNA molecules are available in Supplementary Methods 1.1.

**Table 1:** Detailed definitions of all features (free energy of the tiRNA monomer (EA), free energy of the tiRNA-tiRNA dimer (EAA), free energy of the tiRNA-UTR dimer (EAB), formation energy of the tiRNA-tiRNA dimer (FAA), formation energy of the tiRNA-UTR dimer (FAB), total seed energy (ETS), intermolecular binding seed energy (EIS), probability availability of UTR (PAU), RBS coverage of length 5 (RBS5), RBS coverage of length 11 (RBS11), paired termini (PT), and the length of the translation inhibiting RNA (tiRNA) (L)) used in the initial *in silico* screening of the tiRNA library for repression of a target untranslated region (UTR). This UTR contains a ribosome binding site (RBS) and controls the coding DNA sequence (CDS) of the reporter protein. All binding probabilities of monomers and dimers are respectively derived from base pairing probability matrices estimated by RNAfold [45] and RNAcofold [46].

Name	Definition of the tiRNA feature
EA	ΔG_tiRNA_; free energy of the tiRNA monomer, calculated using RNAfold [45].
EAA	ΔG_tiRNA-tiRNA_; free energy of the tiRNA-tiRNA dimer, calculated using RNAcofold [46].
EAB	ΔG_tiRNA-UTR_; free energy of the tiRNA-UTR dimer, calculated using RNAcofold [46].
FAA	Formation energy of the tiRNA-tiRNA dimer; ΔG_tiRNA-tiRNA_—2 ΔG_tiRNA_.
FAB	Formation energy of the tiRNA-UTR dimer; ΔG_tiRNA-UTR_—ΔG_tiRNA_—ΔG_UTR_. With ΔG_UTR_ defined as free energy of the UTR (including first 50 nucleotides of the CDS RNA), calculated using RNAfold [45].
ETS	Average minimal total energy (gains from intermolecular binding and needs to ‘open’ the binding site) for the binding of unbound parts of the tiRNA monomer to the target UTR, calculated using RNAup [49] for 100 suboptimal structures randomly drawn from Boltzmann ensemble [48]
EIS	Average minimal energy gained from intermolecular binding of unbound parts of the tiRNA monomer to the target UTR, calculated using RNAup [49] for 100 suboptimal structures randomly drawn from Boltzmann ensemble [48]
PAU	Weighted average of the relative number of unbound nucleotides in the UTR monomer with the relative number of nucleotides bound by the tiRNA molecule in the tiRNA-UTR dimer complex as weights.
RBS5	The RBS coverage (relative number of bound nucleotides) in the region C_RBS_−2 to C_RBS_+2, where C_RBS_ is defined as the weighted average of the nucleotides in the UTR bound by the 16S rRNA.
RBS11	The RBS coverage (relative number of bound nucleotides) in the region C_RBS_−5 to C_RBS_+5, where C_RBS_ is defined as the weighted average of the nucleotides in the UTR bound by the 16S rRNA.
PT	Average number of bound nucleotides between the first and the second half of the a tiRNA sequence calculated for 100 suboptimal structures randomly drawn from Boltzmann ensemble [48].
L	The length of the tiRNA (nt)

### Statistical calculations and experimental design

All statistical calculations and analyses were performed in R. Unless otherwise stated, error bars represent the standard deviation (n = 3). All coefficient of determinations (R^2^s) were calculated using the hydroGOF package in R.

#### Experimental design

The 2^6-2^ fractional factorial design, which comprises solely UTR_1_, was generated using the R package FrF2 [50]. In the DOE, the -1 and 1 state of the factors were defined as the 0.1 and 0.9 p-quantiles of the original feature distribution, respectively. The center points are designed to be [0, 0, 0, 0, 0, 0], where 0 represents the average of the absolute values of the tiRNA features in the initial library of 1,500,000 possible tiRNA candidates. For each feature in the experimental design all data points (X_i_) were centered and scaled based on the 0.1 and 0.9 p-quantiles (q_X,0.1_ and q_X,0.9_) of the original distribution of feature X (Eq 3).

$$\tilde{X_{i}}=\frac{X_{\text{i}}-(q_{\text{X},0.9}+q_{\text{X},0.1})/2}{(q_{\text{X},0.9}-q_{\text{X},0.1})/2}$$ (3)

The centered features $\tilde{X}$ were only used in the analysis of the DOE. All data points of the 2^6-2^ fractional factorial design are shown in Table D in S1 Text. The features FAB and EIS were multiplied by -1 to obtain positive regression coefficients as these were expected to be negatively correlated with tiRNA performance.

### Regression models

#### Ordinary least squares (OLS) regression

The OLS regression was done in R. The OLS regression model was calibrated using the absolute (unprocessed) values of FAB for all data points, including data from target UTR_2_. Eq 4 depicts the linear relationship obtained from the OLS regression, where Y_j_ is the relative protein expression when tiRNA j is present, X_j,1_ is feature FAB of tiRNA j, *β*_0_ and *β*_1_ are regression coefficients and *ϵ*_j_ is an error term.

$${\text{log}}_{10}\left(Y_{\text{j}}\right)=\beta_{0}+\beta_{1}X_{\text{j},1}+\epsilon_{\text{j}}$$ (4)

#### Partial least squares (PLS) regression

The PLS regression was done in R with the package pls [51]. The PLS model was validated by splitting the data set from UTR_1_ and UTR_2_ in a training set and validation set (5:1 ratio). Subsequently, the training set was used to create the model by leave-one-out cross validation where predictors were scaled prior to regression (by dividing each variable the sample standard deviation). In PLS regression the matrix of predictors **X** is decomposed into orthogonal score matrix **T** (projection of **X**) and loadings matrix **P**, circumventing potential collinearities in the data set:

X=TP (5)

Subsequently, **Y** is regressed on the scores **T** (and not **X**). The specific PLS algorithm used is kernel PLS, which was described by Dayal et al [52].

## Results and discussion

**Fig 1:**
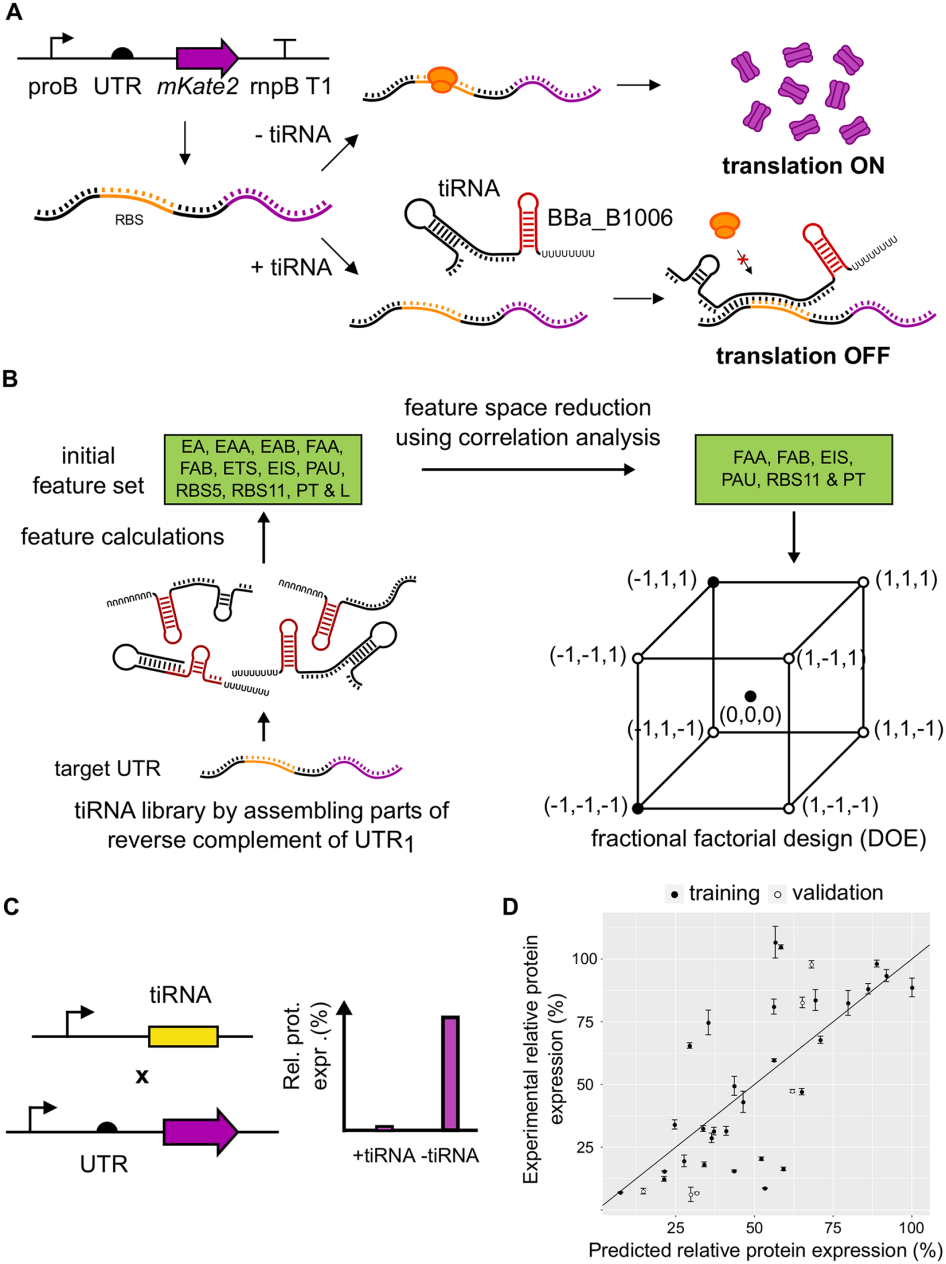
A) Schematic overview of the translation inhibiting RNA (tiRNA) working mechanism B) Workflow for the *in silico* selection of the tiRNAs comprising the design of experiments (DOE) to unravel design principles. The defined tiRNA features (free energy of the tiRNA monomer (EA), free energy of the tiRNA-tiRNA dimer (EAA), free energy of the tiRNA-UTR dimer (EAB), formation energy of the tiRNA-tiRNA dimer (FAA), formation energy of the tiRNA-UTR dimer (FAB), total seed energy (ETS), intermolecular binding seed energy (EIS), probability availability of UTR (PAU), RBS coverage of length 5 (RBS5), RBS coverage of length 11 (RBS11), paired termini (PT), and the tiRNA length (L)) are calculated for a tiRNA library created based on a specific target 5’ untranslated region (UTR). C) *In vivo* assessment of the tiRNA in the designed experiment D) Linking features to tiRNA performance through modeling. The fluorescence (FP) per optical density (OD) for a strain was calculated as follows: (FP/OD)_corrected_ = (FP-FP_bg_)/(OD-OD_bg_) with FP_bg_ = fluorescence of the strain without fluorescent protein expression and OD_bg_ = OD of the medium. The relative protein expression (%) was defined as the (FP/OD)corrected in presence of the riboregulator divided by the (FP/OD)corrected in the absence of the riboregulator.

The *trans* expressed tiRNAs are *de novo* designed to inhibit translation initiation of a gene of interest, the rate-limiting step in translation [53], as depicted in Fig 1A. Contrary to previous efforts to construct repressing riboregulators, these RNA devices are constructed from scratch without a functional chassis, which is often based on a natural occurring RNA regulation device [26, 54, 55]. To enable reliable forward engineering of tiRNAs, a workflow to improve the *de novo* development of repressing riboregulators through DOE guided exploration of the sequence space was developed and optimized (see Fig 1B–1D). First, possibly important features for translational inhibiting riboregulators are derived from literature. Secondly, the number of features are reduced by removing correlations. Subsequently, this reduced set of tiRNA properties is used in an experiment designed to unravel design principles to build effective tiRNA molecules. In the DOE, tiRNAs are constructed that explore the feature space in an intelligent way. Ultimately, thoroughly analyzing the performance of the constructed tiRNAs with varying features can improve the knowledge on the structure-function relationship, which correlates to better predictability of *de novo* created riboregulators [26, 33, 36].

### Identification of determinative features of repressing riboregulators

**Fig 2:**
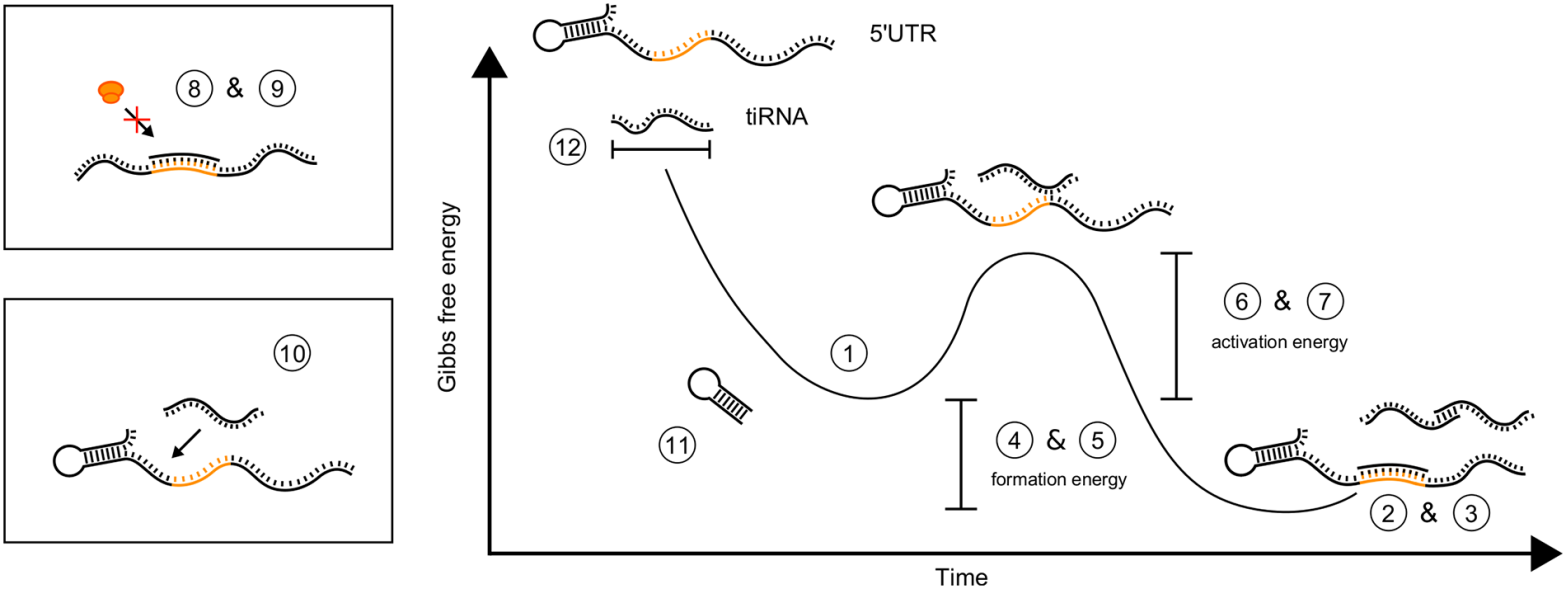
Schematic overview of all potentially determinative features of translation inhibiting RNAs (tiRNAs): 1) free energy of the tiRNA monomer (EA), 2) free energy of the tiRNA-UTR dimer (EAB), 3) free energy of the tiRNA-tiRNA dimer (EAA), 4) formation energy of the tiRNA-UTR dimer (FAB), 5) formation energy of the tiRNA-tiRNA dimer (FAA), 6) intermolecular binding seed energy (EIS), 7) total seed energy (ETS), 8) RBS coverage of length 5 (RBS5), 9) RBS coverage of length 11 (RBS11), 10) probability availability of UTR (PAU), 11) paired termini (PT), and 12) tiRNA length (L).

In total, 12 potentially determinative features of efficient tiRNA were identified and derived from literature (see Fig 2 and Table 1 for more details). These 12 features represent all design principles previously used in riboregulator construction. Five out of the 12 indentified features are based on structural properties. Namely, two features are defined to quantify RBS coverage, i.e. RBS5 and RBS11, which is the average base pairing probability in the region of the RBS with length 5 and 11, respectively. The third feature quantifies the amount of paired termini (PT) and the availability of the UTR is evaluated by the PAU property. The last structural feature is determined by the length of the tiRNA (L). The remaining seven defined features are based on properties relating to thermodynamics. The energy required for the formation of the tiRNA-tiRNA dimer and the tiRNA-UTR dimer are defined as FAA and FAB, respectively. These formation energies are calculated based on the estimated Gibbs free energy of the final dimer and both initial monomer states, which are described by the EA, the EAA, and the EAB features. In addition, two features describe the activation energy: intermolecular binding seed energy (EIS) and the total seed energy (ETS). Notably, most previous approaches to specify design rules for riboregulators only take the MFE structures into account, simplifying the Boltzmann ensemble of RNA secondary structures and the corresponding complex dynamic energy landscape of regulatory RNAs [56]. The workflow followed here to improve the *de novo* development of repressing riboregulator through DOE guided exploration of the sequence space is depicted in Fig 1B. Here, simplifications were minimized by taking the Boltzmann ensemble into account as much as possible.

### Feature space reduction using correlation analysis

A library of 1,500,000 unique possible tiRNA sequences with length 20, 30 or 40 nucleotides (nt) was created *in silico* based on UTR_1_ (see Table E in S1 Text) using custom perl code. To generate this library, sequences were generated by combining a random number of different (randomly chosen) parts (length >= 2 nt) of the reverse complement of UTR_1_ and keeping the order of occurrence of these different parts, as effective riboregulators typically contain parts of the reverse complement of the target UTR.

**Fig 3:**
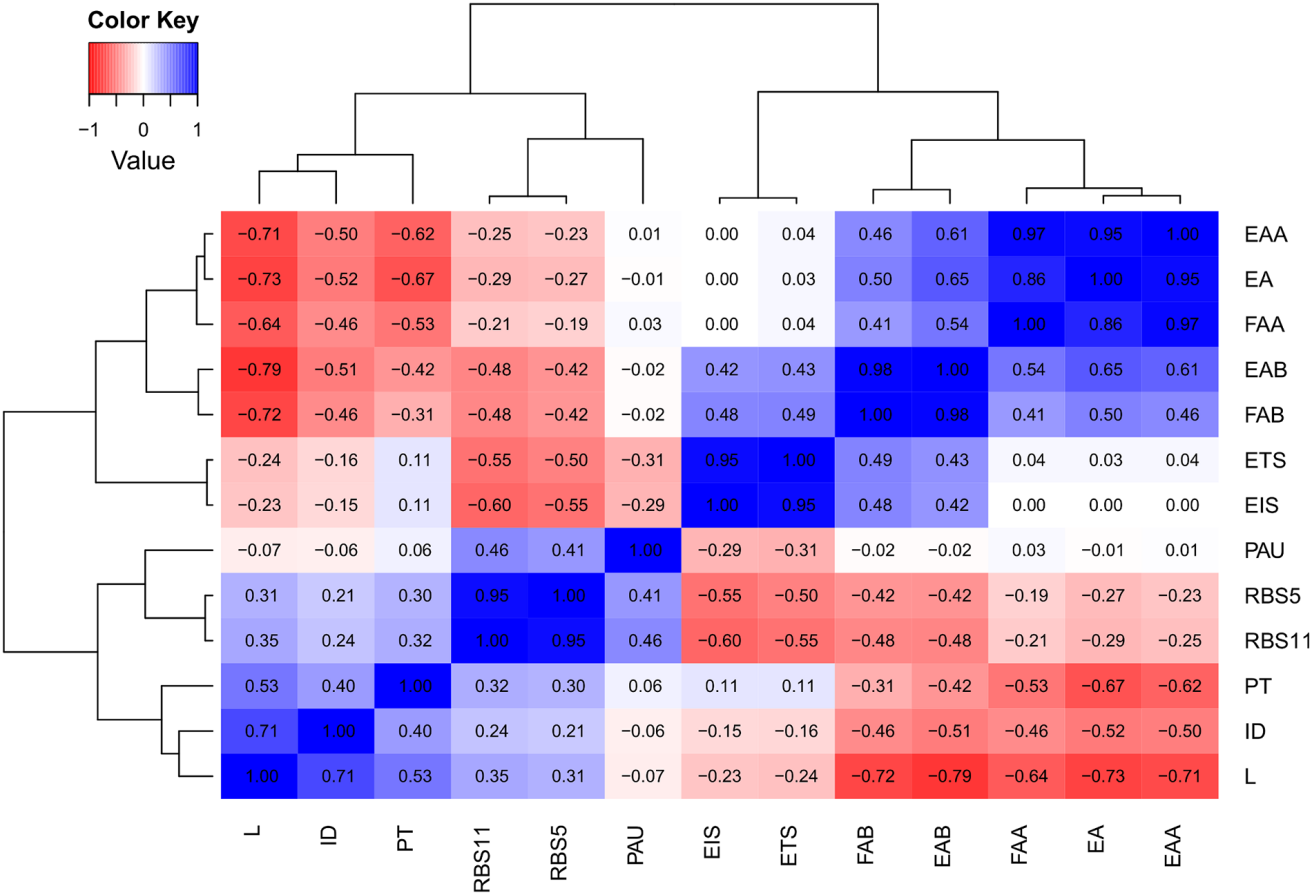
Heatmap of the Pearson correlation coefficients (PCCs) between all features of the translation inhibiting RNA (tiRNA) library, with L = length, EA = free energy of the tiRNA monomer, EAA = free energy of the tiRNA-tiRNA dimer, EAB = free energy of the tiRNA-UTR dimer, FAA = formation energy of the tiRNA-tiRNA dimer, FAB = formation energy of the tiRNA-UTR dimer, ETS = total seed energy, EIS = intermolecular binding seed energy, PAU = probability availability of UTR, RBS5 = RBS coverage of length 5, RBS11 = RBS coverage of length 11, and PT = paired termini (see Table 1 and Fig 2). The fluorescence (FP) per optical density (OD) for a strain was calculated as follows: (FP/OD)_corrected_ = (FP-FP_bg_)/(OD-OD_bg_) with FP_bg_ = fluorescence of the strain without fluorescent protein expression and OD_bg_ = OD of the medium. The relative protein expression (%) was defined as the (FP/OD)corrected in presence of the riboregulator divided by the (FP/OD)corrected in the absence of the riboregulator.

**Fig 4:**
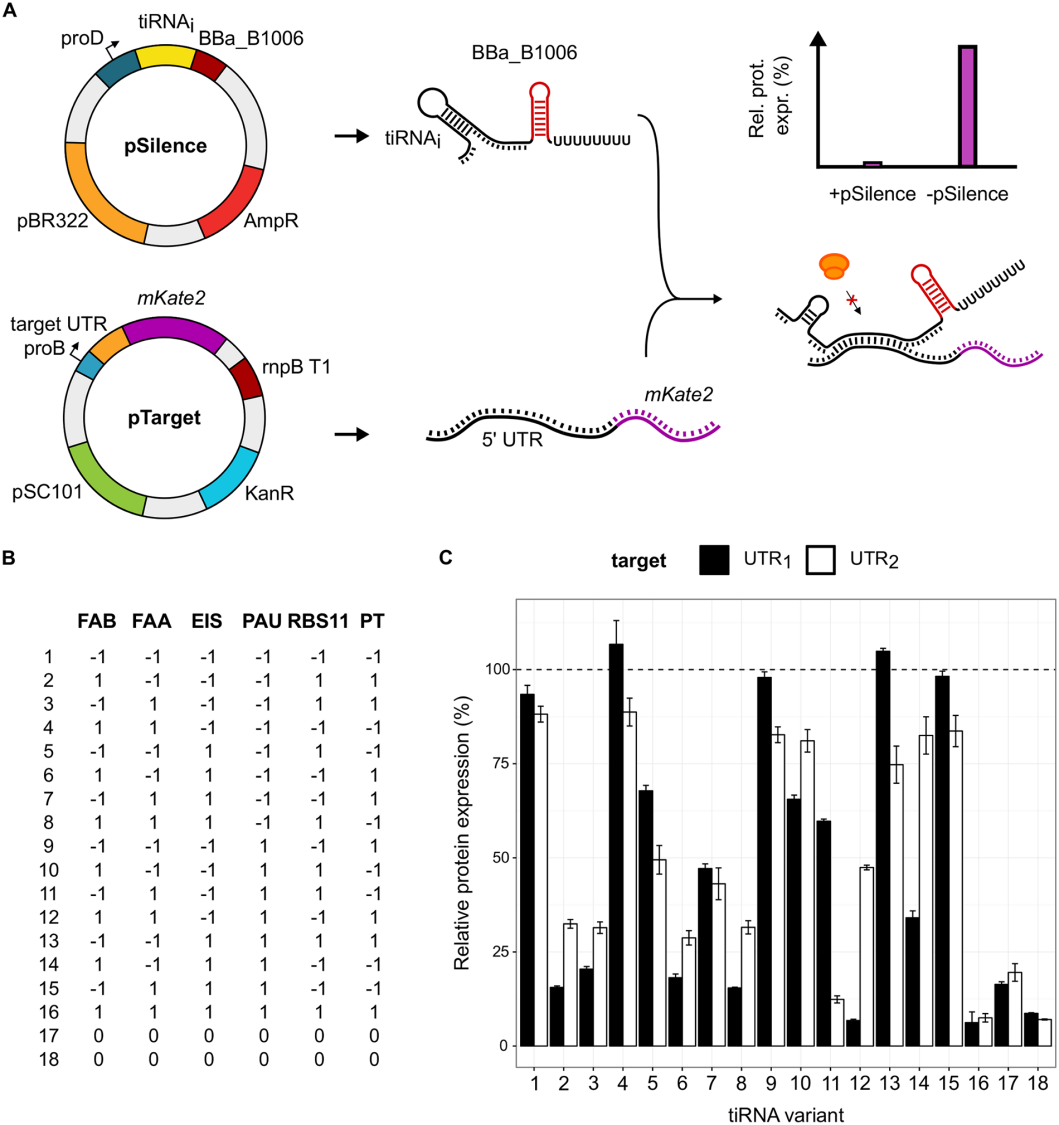
The results from the designed experiment to unravel the principles for translation inhibiting RNA (tiRNA) design. (A) The practical execution of the design of experiments (DOE). All tiRNAs, representing a data point in the DOE, are coexpressed with the target untranslated region (UTR) and the riboregulator efficiency is determined. (B) All tiRNAs with corresponding feature data point in the DOE, where the features are calculated using UTR_1_. (C) The tiRNA performance, expressed in relative protein expression, where lower expression represents more effective tiRNAs. The performance was evaluated using both UTR_1_ and UTR_2_. The 100% relative protein expression represents the protein expression in absence of the tiRNA. Error bars represent standard deviation (n = 3).

The amount of correlations between the various features was reduced by analyzing existing correlations between all features, and subsequently removing correlations above a set threshold of 0.75. This was done by calculating the Pearson correlation coefficients (PCCs) (see Fig 3). The correlations between FAA, EA, and EAA, between FAB and EAB, and between EIS and ETS are caused by one or more features being used in the calculation of another feature. Also, RBS5 and RBS11 are correlated, which can be explained by the fact that the RBS region covered by RBS5 is also covered by RBS11. Finally, the length of tiRNA (L) is correlated with EAB as the stability of the tiRNA-UTR complex increases (lower Gibbs free energy) with the tiRNA length. The feature space was reduced, while minimizing information loss, by removing correlations between various features. To this end, one feature of a set of correlated features was selected (|PCC| > 0.75). The reduced set of the tiRNA features X_i_ with limited correlations, i.e. FAA, FAB, EIS, PAU, RBS11, and PT, was used in a DOE to unravel the features with the most influence on the repression efficiency of these pure riboregulators (see Fig 1B). More specifically, a fractional factorial 2-level design was used with a resolution of IV (2^6-2^ design), comprising two center points and 16 factorial points. After rescaling all features (see Section and Table D in S1 Text for details), the 18 best suiting data points were selected from the library of 1,500,000 tiRNA candidates. The density of all tiRNA features of the complete constructed library with the 0.1 and 0.9 p-quantiles is depicted in Figure D in S1 Text. Because the features are calculated based on the sequence of a generated tiRNA candidate, the factors cannot be set to a specific value. Instead, suitable sequences were selected from the tiRNA candidate library based on the residual sum of squares (RSS) between the real data point of the experimental design and the actual features a this specific candidate (overall, average RSS is 0.59). The selected tiRNA sequences (one feature was selected from features with a PCC above 0.75) with their corresponding theoretical data point are depicted in Table A in S1 Text and Fig 4A, respectively.

### *In vivo* analysis of tiRNA performance

Subsequently, the performance of these 18 tiRNAs in the DOE was evaluated *in vivo* as depicted in Fig 4A. In this experimental setup, the tiRNA molecules are expressed from pSilence plasmids carrying the pBR322 ori, which have an approximately fourfold higher copy number compared to the pSC101 ori of the pTarget plasmids utilized for UTR expression [37], and are under the control of the proD promoter, which showed 8.4 fold higher transcriptional activity compared to the proB promoter used for UTR expression [38]. The overall higher relative tiRNA expression (compared to its target UTR) was chosen based on the fact that *trans* acting sRNA typically require relatively higher expression of the sRNA compared to its target, in both natural and synthetic sRNA regulation systems [57, 58].

To enlarge the data set, the pSilence plasmids were co-transformed with the pTarget plasmid containing UTR_1_ or UTR_2_ (a truncated version of UTR_1_, see Table E in S1 Text), respectively, evaluating the repression efficiency of the tiRNAs in the DOE. Compared to UTR_1_, UTR_2_ results in 3.3 times less production of fluorescent protein in absence of any riboregulator (see Figure E in S1 Text) although the thermodynamic stability of UTR_1_ is much higher than UTR_2_ (-27.6 and -17.3 kcal/mol, respectively), which is in contrast to previous studies inversely relating translation to the Gibbs free energy of the UTR [59, 60]. Moreover, the UTR_2_ forms much less base pairs in the region around the Shine-Dalgarno (SD) sequence (see Figure E in S1 Text), making the RBS possibly more accessible. However, the removal of a terminal stem loop in the 5’ UTR could decrease mRNA stability by exposing the RNA to RNases, resulting in a decrease in fluorescence [61, 62].

The outcome of the designed experiment is depicted in Fig 4C. In this DOE tiRNA_1_ ([-1,-1,-1,-1,-1,-1]) does not possess any of the features and serves as a control when combined with UTR_1_ to determine the burden of the promoter used, which is not significantly different from the strain containing pBlank_1_ and pTarget_1_. This is in accordance with literature determining the burden of RNA riboregulators, which is low compared to other types of gene expression regulation [21]. The activity of the *de novo* designed riboregulators shows that almost all tiRNAs were active. Specifically, eight of the 18 tiRNAs targetting UTR_1_ inhibit the translation initiation of UTR_1_ with more than 75%. The most repressing tiRNAs reduce protein expression of UTR_1_ to about 6% of the original expression level. The highest dynamic range of translation repression among all data points is 16, which is higher than previously described repressing riboregulators [26]. Moreover, these tiRNA are created *de novo*, without using a naturally occurring functional chassis.

Overall, the repression levels on UTR_2_ are comparable to those of UTR_1_, indicating that the truncated part distal to the RBS BBa_B0032 is less important for riboregulator activity. There is a clear difference in repression efficiency between tiRNA_1_ ([-1,-1,-1,-1,-1,-1]) and tiRNA_16_ ([1, 1, 1, 1, 1, 1]), showing the importance of at least one of the selected features for translational inhibition. Another interesting fact is the high repression rate of tiRNA_17_ and tiRNA_18_, which are the center points of the DOE. The good performance of these center points indicate that choosing less extreme values can also result in effective translational repression.

### Linking features to tiRNA activity

**Fig 5:**
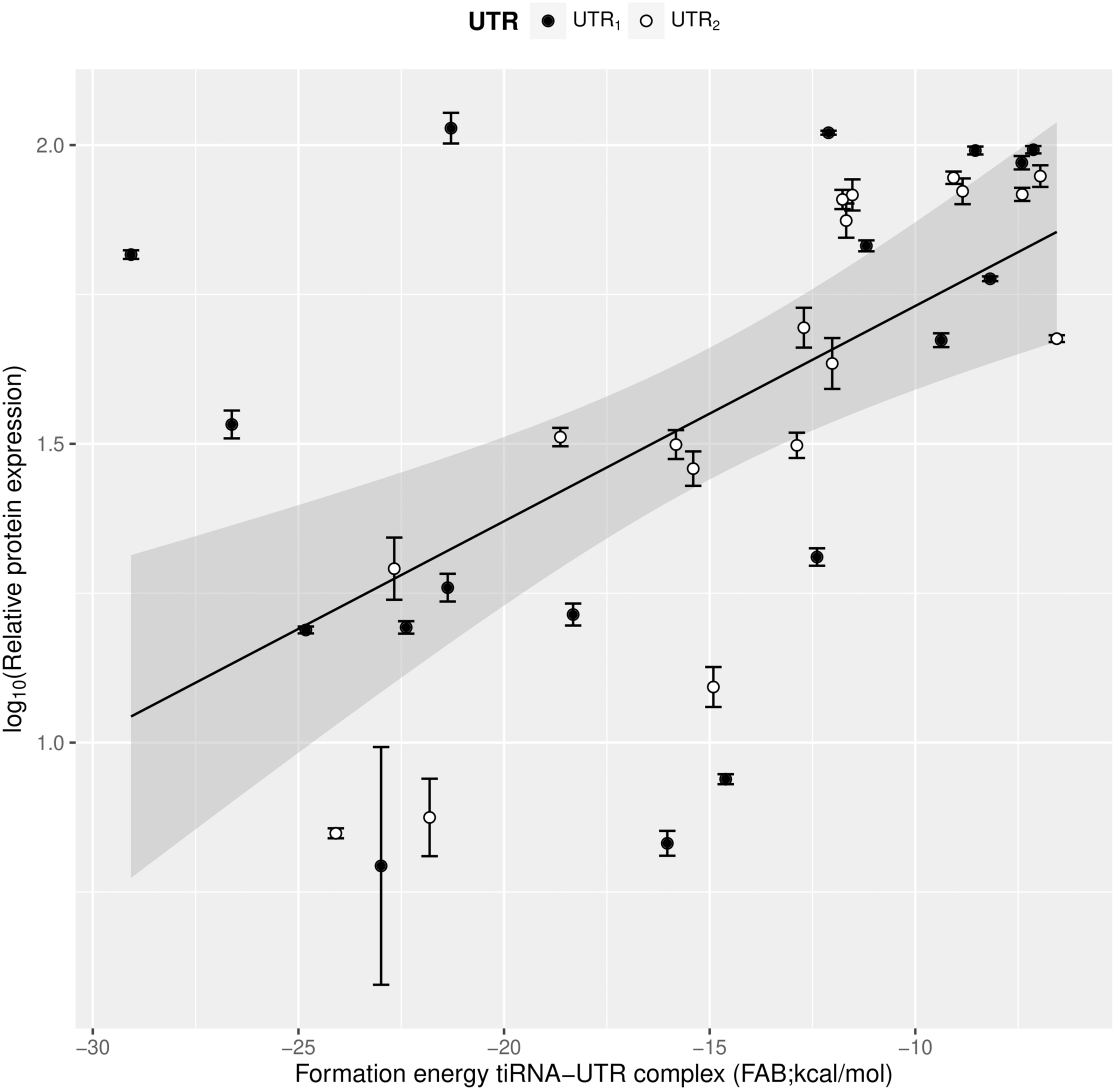
Plot of the ordinary least squares (OLS) regression of the linear model, linking log_10_ of the relative protein expression to the translation inhibiting RNA (tiRNA) feature formation energy of the tiRNA-UTR dimer (FAB). All data points were used, including the effect of the tiRNAs on both untranslated region 1 (UTR_1_) and UTR2. The gray area depicts the 95% confidence interval of the OLS linear regression. Error bars represent the standard deviation (n = 3).

To unravel underlying design principles of repressing tiRNAs, an OLS linear regression analysis was performed in a first approach. To this end, a linear regression model was applied using all data points in the experimental design. All relative expression percentages from all data points of the experimental design are plotted against all normalized features (with only UTR_1_ as target) in Figure F in S1 Text. The predicted MFE secondary structure of the 18 different tiRNA:UTR complexes for UTR_1_ and UTR_2_ is depicted in Figure G and H in S1 Text, respectively. Relative expression percentages plotted against all absolute features for all data (including the repression percentages of UTR_2_) are depicted in Figure I in S1 Text. Only two factors in the linear model had a significant influence, namely FAB (p < 0.05) and PT (p < 0.1). Factors FAB and PT also had significant influence on several other reported riboregulator systems with or without the aid of Hfq [9, 18, 26, 32, 33, 36]. When using only these two features in a linear regression model, only the factor FAB was significant (p < 0.05), while factor PT turned out to be not significant (p > 0.1). As the factor FAB is based on thermodynamic properties, it was hypothesized that the relation between FAB and the relative protein expression is exponential. Therefore, a linear model was used to relate the logarithmic of relative protein expression percentage to the tiRNA feature FAB (see Eq 4). The outcome of this OLS regression is depicted in Fig 5. Despite the significant influence of FAB in the DOE, this basic model is still unable to explain all tiRNA functionality which is reflected by the fact that the majority of the data points are not within the 95% confidence interval of the OLS model.

Data driven approaches using regression methods have previously been successful in biological engineering [63, 64] and, more specifically, forward design of various RNA devices [26, 36, 65]. Therefore, in a second approach, PLS regression was performed. To maximize the information possibly linked to tiRNA activity, the 12 defined features were included.

**Fig 6:**
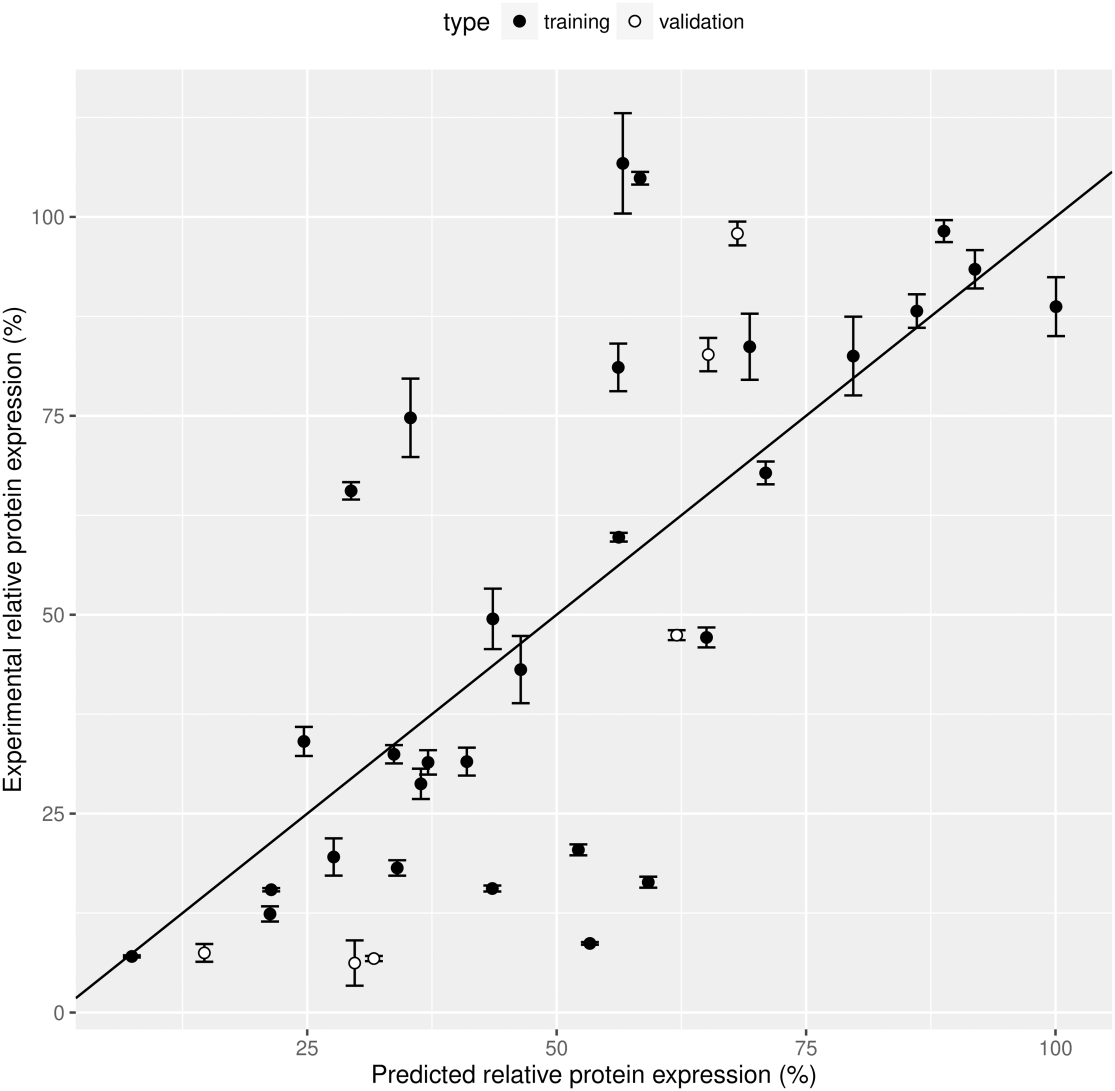
Validation of partial least squares (PLS) regression model predicting relative protein expression from 12 predictors (features of translation inhibiting RNA (tiRNA)). Plot of experimental versus predicted relative protein expression via PLS model for the training set (used for model calibration) and the validation set (used to test the model efficiency; coefficient of determination (R^2^) equal to 0.69). Error bars represent the standard deviation (n = 3).

To perform the PLS regression, all data points from UTR_1_ and UTR_2_ were split into two subsets: one set used for model calibration, i.e. training set, and one independent set used for model validation, i.e. test set. The latter set was selected by randomly picking tiRNAs from three groups which are ordered based on the averaged gene expression of both UTR_1_ and UTR_2_. The test data comprised tiRNA_9_, tiRNA_12_, and tiRNA_18_, all other tiRNAs were used in the training set. Before regression, the absolute values of the tirna features were scaled through division by the sample standard deviation. Model calibration was done using all 30 data points from the training set and uses 12 features (k = 12) describing tiRNA performance. The final model contained 4 latent variables and was selected based on the root mean squared error of prediction and the explained Y variance. By using the training set, a final PLS model contains 63.9% of the X variance, which explained 50.4% variance of the response variable and a R^2^ (describing the model efficiency) of 0.50 (see Fig 6). To validate this PLS model, the independent validation set was used to assess the quality of the PLS model. The R^2^ of this validation set was 0.69, indicating that the model successfully explains tiRNA activity. To identify the most important factors in the PLS regression model, all estimated regression coefficients are calculated (see Table F in S1 Text for all coefficients and scaling factors). The regression coefficients of the 12 tiRNA features are shown in Figure J in S1 Text. The cumulative loadings of the 4 components and the biplot of the first two components are depicted in Figure K and L in S1 Text, respectively. From these estimates the formation energy of the UTR-tiRNA complex is again inversely correlated to the final protein expression as both regression coefficients of EAB and FAB are positive. This link between dimer stability and riboregulator performance was also previously observed in other RNA devices [26, 33]. Other observations are the negative relation between FAA and protein expression, indicating that a stable tiRNA-tiRNA dimer complex decreases tiRNA efficiency. Besides these thermodynamic factors, structural features PAU and PT are inversely correlated to protein expression. Thus, as described in literature [29, 32, 35], target nucleotide availability and the number of paired termini (linked to RNA stability) in the riboregulator monomer is important for repression efficiency. Contrary to previous studies, activation energy and total RBS occlusion has a rather limited influence on gene repression.

Overall, the PLS modelling approach employed here successfully predicts tiRNA activity based on the described 12 features, which were defined based on literature. However, various features used in previously described efforts were quantified using different methods [26, 33]. This lack of standardized methods to determine thermodynamic and structural features of riboregulators complicates forward engineering of riboregulators. Also, the diverse range of features required to explain tiRNA functionality is an indication of the complex nature of the regulatory mechanism of riboregulation. As such, RNA regulation might require properties unknown today, which can be discovered using recently developed technologies allowing detailed structural analysis of riboregulators with a high-throughput. For instance, SHAPE-Seq allows *in vivo* characterization of RNA structure by coupling chemical probing techniques to next-generation sequencing technology [66, 67].

### Conclusions

The developed approach allows *de novo* design of translation inhibiting riboregulators, which further broadens the RNA regulator toolbox. From the 18 constructed tiRNAs molecules designed in the DOE eight tiRNAs repressed protein production with more than 75%. The riboregulators described in this paper do not require any coexpressed proteins, which increases their applicability to build complex genetic circuitry. For instance, it allows to reconstitute a RNase III site (resulting in RNA degradation [68]) or interference with guide RNAs of a CRISPR system to obtain complex biological functions. To further improve riboregulator design several basic modelling approaches were employed. However, these basic efforts were unable to fully explain tiRNA performance, indicating the complexity of riboregulator repression. Previous efforts often rely on several criteria to engineer riboregulators of various types with varying success [26, 33, 36, 69]. Based on these efforts, 12 features were defined and used in a DOE to explore the tiRNA feature space. Subsequently, to improve the reliability of *de novo* forward engineering of repressing riboregulators, a sequence-function model was constructed to link tiRNA functionality to the defined tiRNA features. To this end, both structural and thermodynamic tiRNA features were used in a PLS regression model, which was evaluated using an independent test set (R^2^ equal to 0.69). The success of this data driven approach indicates the importance of machine learning techniques in modern synthetic biology to grasp the ever increasing complexity of biological design. Furthermore, the complex nature of riboregulation and the limited knowledge of the underlying working mechanisms makes engineering RNA devices challenging. To this end, novel technologies (for instance SHAPE-Seq) enable high-throughput study of the structure-function relationship of various types of riboregulators in detail by combining RNA structural probing techniques and next-generation sequencing technology, allowing better prediction of riboregulator performance [66, 67].

## Supporting information

S1 Text A single pdf file containing all supplementary methods, figures, and tables. (PDF)
