# Supplementary material for: Exploring of the feature space of de novo developed post-transcriptional riboregulators
Source: PLoS Comput Biol. 2018 Aug 17;14(8):e1006170. doi: 10.1371/journal.pcbi.1006170 (PMC6114898; doi:10.1371/journal.pcbi.1006170)
Supplement: S1 Text — (PDF) [file pcbi.1006170.s001.pdf]

Exploring the feature space of *de novo* developed post-transcriptional riboregulators

Gert Peters[1], Jo Maertens[1], Jeroen Lammertyn[2], Marjan De Mey[1*]

1 Centre for Synthetic Biology, Ghent University, Coupure Links 653, B-9000 Ghent, Belgium
2 BIOSYST-MeBioS, KU Leuven, Willem de Croylaan 42, B-3001 Leuven, Belgium

* Marjan.DeMey@UGent.be

Supporting information

1 Supplementary Methods

1.1 Quantification of thermodynamic properties

In general, complex formation energy $\Delta G^{ij}{}_{form}$ can be calculated as follows:

$$\Delta G^{ij}{}_{form} = \Delta G^{ij}{}_{dimer} - (\Delta G^{i}{}_{monomer} + \Delta G^{j}{}_{monomer}) \quad (1)$$

Where $\Delta G^{ij}{}_{dimer}$, $\Delta G^{i}{}_{monomer}$ and $\Delta G^{j}{}_{monomer}$ are the estimated Gibbs free energy of the final dimer, and both initial monomer states, respectively. The features describing thermodynamic properties of translation inhibiting RNA (tiRNA) are free energy of the tiRNA monomer (EA), free energy of the tiRNA-tiRNA dimer (EAA), free energy of the tiRNA-UTR dimer (EAB), formation energy of the tiRNA-tiRNA dimer (FAA) and formation energy of the tiRNA-UTR dimer (FAB) (see Table 1).

1.2 Quantification of activation energy

The activation energy is estimated by the hybridization energy of consecutively unbound nucleotides of the tiRNA complex, the seed region, with the untranslated region (UTR). To get a good representation of the Boltzmann ensemble a random sample of 100 suboptimal structures of the tiRNA molecule are drawn with probabilities equal to their Boltzmann weights using the RNAsubopt algorithm [1]. This is done via stochastic backtracking in the partition function using RNAsubopt [1]. The intermolecular binding between the unbound part of the antisense and the UTR is estimated using the RNAup algorithm, which first calculates the energy requires to 'open' the binding site and subsequently calculates the minimal energy gained from intermolecular binding [2]. This is done for all possible unbound sequences with a length between two and six nucleotides of the tiRNA monomer. Two features are calculated for each of the 100 suboptimal structures: EIS and ETS. To get a general feature for a tiRNA molecule the average is calculated of the minimal intermolecular binding seed energy (EIS) and total seed energy (ETS) of the 100 structures.

1.3 Calculation of structural tiRNA features

Besides these previously mentioned thermodynamic features, some structural properties were previously described to improve translation initiation repression using solely RNA interactions.

UTR availability One possible determinative feature relates to the availability of the target region in the mRNA, which was previously used as design principle for silencing small RNAs (sRNAs) as the mRNA is not in a constant state but rather in a state of constant structural fluctuation near minimum free energy (MFE) structures[3–6]. To account for a certain volatility of regions in the mRNA, Johnson et al[3] analyzed suboptimal mRNA structures to find regions which alter their structure without significantly changing the Gibbs free energy of the global structure. These regions are assumed to be more accessible and matched naturally occurring antisense target sites[3]. In the design of experiments (DOE) the availability of the UTR nucleotides are accounted for by the $\mathrm{P}_{\text{availability}}$ term, probability availability of UTR (PAU), which is calculated based on the partition function of the tiRNA-UTR dimer and the UTR monomer. For each nucleotide of the UTR the availability (relative number of unbound nucleotides) in the UTR monomer complex ($\mathrm{P}_{\text{UTR,available}}$) and the coverage (relative number of nucleotides bound by the antisense molecule) in the tiRNA-UTR dimer complex ($\mathrm{P}_{\text{UTR,tiRNA-coverage}}$) is determined. Based on these two terms the overall availability term (PAU; $\mathrm{P}_{\text{availability}}$) is determined (weighted average of $\mathrm{P}_{\text{UTR,available}}$ with $\mathrm{P}_{\text{UTR,tiRNA-coverage}}$ as weights).

$$P_{\text{availability}} = \frac{\sum_0^{n_{\text{UTR}}} P_{\text{UTR,available}} \times P_{\text{UTR,tiRNA-coverage}}}{\sum_0^{n_{\text{UTR}}} P_{\text{UTR,tiRNA-coverage}}} \tag{2}$$

ribosome binding site (RBS) coverage To quantify the RBS coverage, two features were defined (RBS coverage of length 5 (RBS5) and RBS coverage of length 11 (RBS11)) as the base pairing probability in the region of the RBS. For the calculation of both features the weighted average of the nucleotides in the UTR bound by the 16S rRNA forms the center of the RBS region. Based on this center the RBS coverage in the regions $\mathrm{C}_{\text{RBS}}-5$ to $\mathrm{C}_{\text{RBS}}+5$ (RBS11) and from $\mathrm{C}_{\text{RBS}}-2$ to $\mathrm{C}_{\text{RBS}}+2$ (RBS5) is calculated based on the partition function estimated by RNAfold[7].

Paired termini (PT) Another structural feature improving translational repression is the presence of paired termini in the silencing RNA molecule, which is strongly related to its thermodynamical stability, resulting in efficient gene silencing[8,9]. The feature PT is calculated by again drawing a random sample of 100 suboptimal structures of the tiRNA molecule with probabilities equal to their Boltzmann weights (using RNAsubopt[1]). From these 100 structures the average number of bound nucleotides between the first and the second half of the tiRNA sequence is calculated.

2 Supplementary Figures

```
CACAGCTAACACCACGTCGTCCCTATCTGCTGCCCTAGGTCTATGAGTGGTTGCTGGA
TAACTTTACGGGCATGCATAAGGCTCGTATAATATATTCATAGTCTTTAGAAAGTTAA
AATTATTAAGGGAAACCTGCCTACTGAAAAAAAAAACCCCGCCCCTGACAGGGCGGGG
TTTTTTTT
```

Fig A. Overview of a DNA construct for translation inhibiting RNA (tiRNA) expression. As an example, the insert of pSilence_1 is displayed with the proD promoter[10], tiRNA_1 riboregulator, and BB_B1006 terminator[11].

```
CTAGAGCACAGCTAACACCACGTCGTCCCTATCTGCTGCCCTAGGTCTATGAGTGGTT
GCTGGATAACTTTACGGGCATGCATAAGGCTCGTAATATATATTCAGGGAGACCACAA
CGGTTTCCCTCTACAAATAATTTTGTTTAACTTTTACTAGAGTCACACAGGAAAGTAC
TAGATGGTTAGCGAGCTGATCAAAGAAAACATGCACATGAAACTGTATATGGAAGGCA
CCGTGAATAACCACCACTTTAAATGTACCAGCGAAGGTGAAGGTAAACCGTATGAAGG
CACCCAGACCATGCGTATTAAAGCAGTTGAAGGTGGTCCGCTGCCGTTTGCATTTGAT
ATTCTGGCAACCAGCTTTATGTATGGCAGCAAAACCTTTATTAACCATACCCAGGGTA
TCCCGGATTTTTTCAAACAGAGCTTTCCGGAAGGTTTTACCTGGGAACGTGTTACCAC
CTATGAAGATGGTGGTGTTCTGACCGCAACCCAGGATACCAGTCTGCAGGATGGTTGT
CTGATTTATAATGTGAAAATTCGCGGTGTGAACTTTCCGAGCAATGGTCCGGTTATGC
AGAAAAAAACCCTGGGTTGGGAAGCAAGCACCGAAACCCTGTATCCGGCAGATGGTGG
TCTGGAAGGTCGTGCAGATATGGCACTGAAACTGGTTGGTGGTGGTCATCTGATTTGC
AATCTGAAAACCACCTATCGTAGCAAAAAACCGGCAAAAAATCTGAAAATGCCTGGCG
TGTATTATGTTGATCGTCGTCTGGAACGTATTAAAGAGGCAGATAAAGAAACCTATGT
GGAACAGCATGAAGTTGCAGTTGCACGTTATTGTGATCTGCCGAGCAAACTGGGTCAC
CGCTGATAACCATGGGCTAGCGGTTTGAAGGGTATTGGTCGGTCAGTTTCACCTGATT
TACGTAAAAACCCGCTTCGGCGGGTTTTTGCTTTTGGAGGGGCAGAAAGATGAATGAC
TGTC
```

Fig B. Overview of a DNA construct for untranslated region (UTR) expression. As an example, the insert of $pTarget_1$ is displayed with the proB promoter[10], UTR_1[10], *mKate2* reporter gene[12], and rnpB T1 terminator[11].

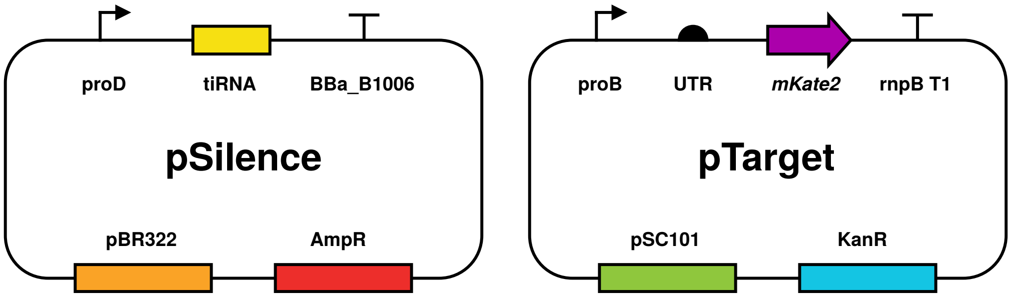

Fig C. Schematic overview of the two plasmid types used in this study. The plasmids pSilence and pTarget are used to respectively express translation inhibiting RNAs (tiRNAs) and the target untranslated regions (UTRs) upstream of the reporter gene *mKate2*[12]. pSilence comprises a medium-copy vector (pBR322 origin of replication and ampicillin resistance marker, originating from pSB6A1[13]) using proD[10] as promoter and BBa_B1006[11] as terminator. pTarget comprises a low-copy vector (pSC101 origin of replication and kanamycin resistance marker) with proB[10] as promoter, *mKate2*[12] as reporter gene, and rnpB T1[11] as terminator. Details of all tiRNAs, plasmids and important DNA sequences used in this study are listed in Table A, B and C, respectively.

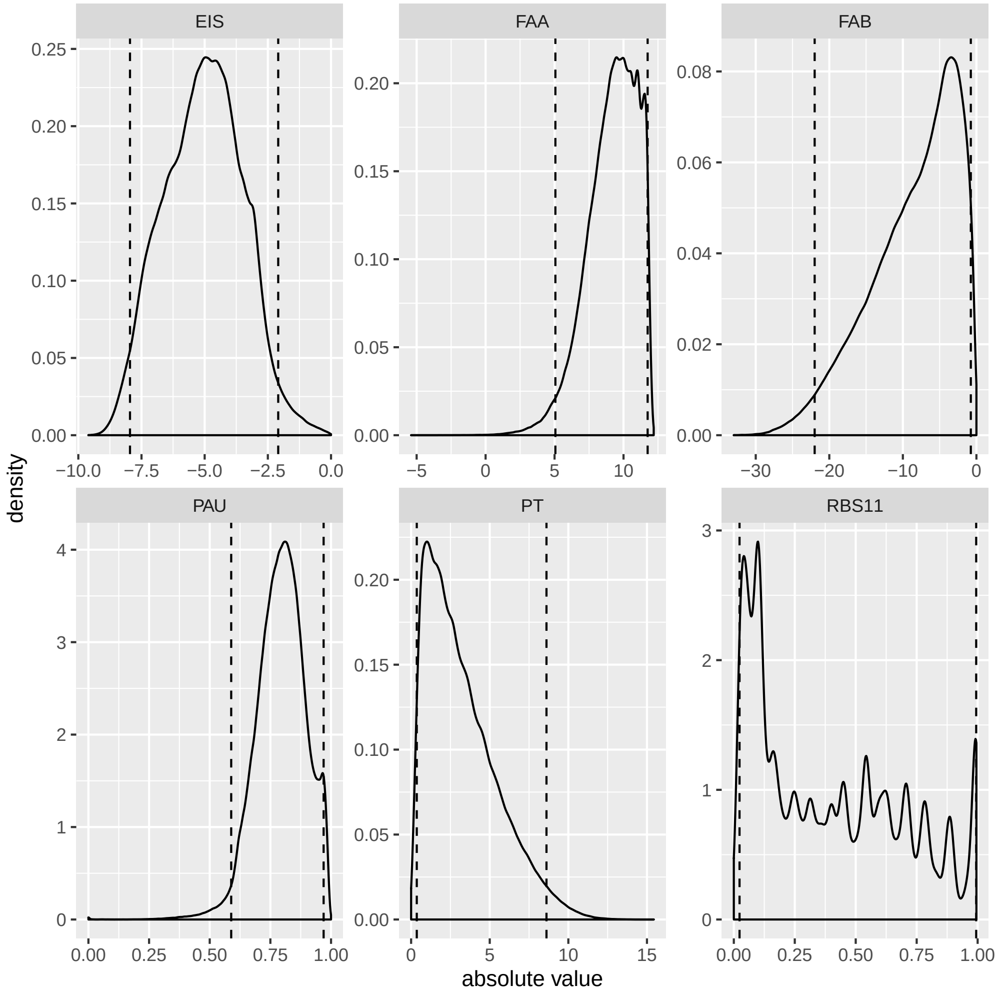

Fig D. Density of all features of tiRNA with the 0.1 (-1 level) and 0.9 p-quantiles (+1 level) indicated as vertical stripped lines.

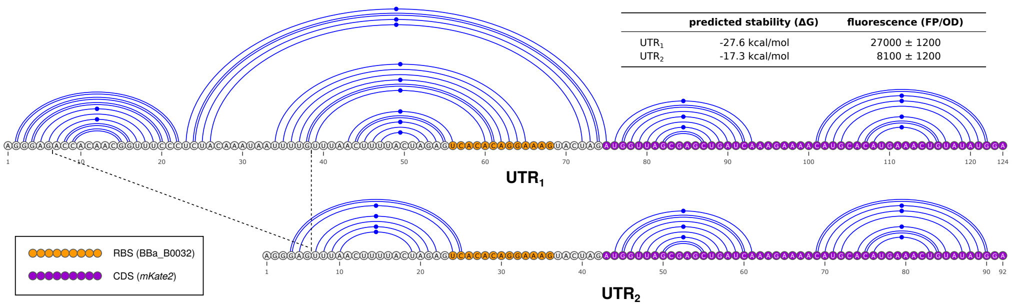

Fig E. Predicted minimum free energy (MFE) structures of the two mRNAs used in this study, comprising the untranslated region (UTR) and the coding DNA sequence (CDS) part. Both UTRs contain the ribosome binding site (RBS) BBa_B0032. The predicted Gibbs free energy (ΔG) is depicted (top right) along with the experimentally determined fluorescence $(FP/OD_{700})_{corrected}$ for both UTR_1 and UTR_2.

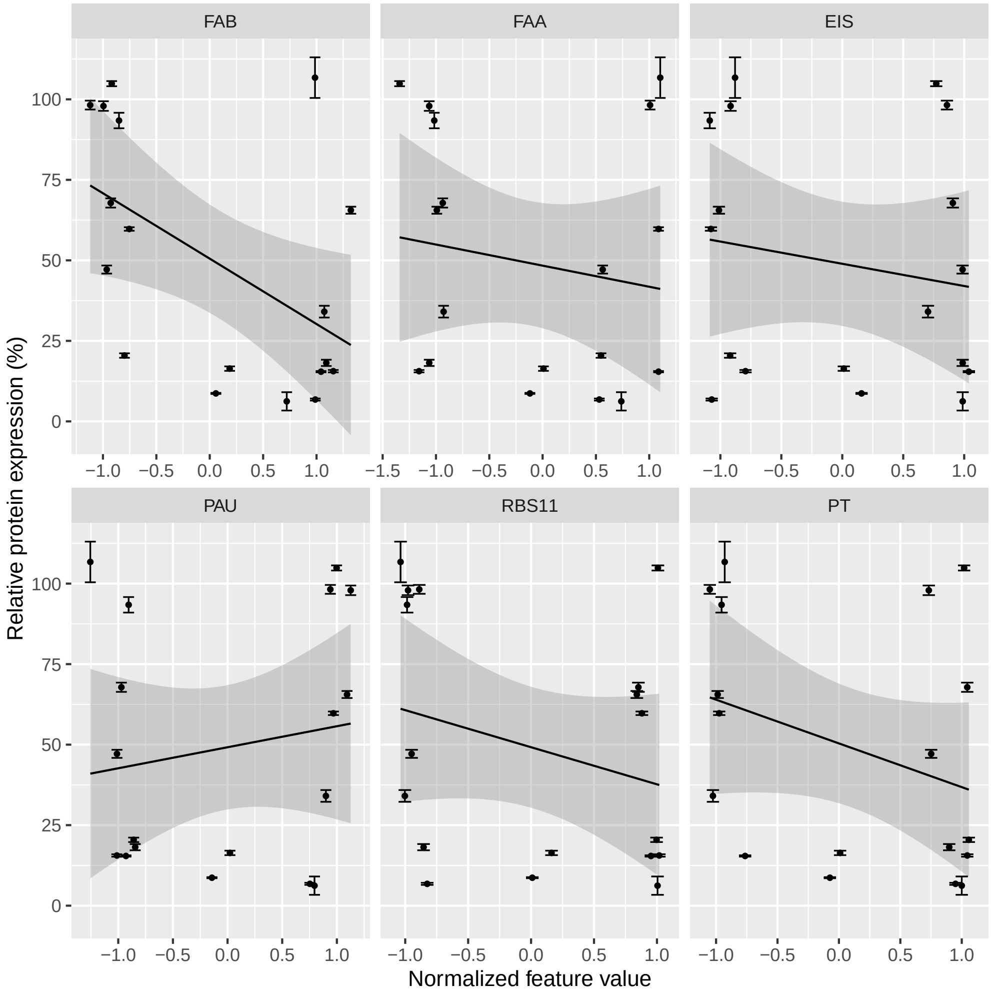

Fig F. Plot of the percentage relative expression of all data points in the experimental design (comprising solely UTR_1) against the normalized translation inhibiting RNA (tiRNA) features of the reduced feature set. The six factors used in the design of experiments (DOE) are the features in the reduced feature set (formation energy of the tiRNA-tiRNA dimer (FAA), formation energy of the tiRNA-UTR dimer (FAB), intermolecular binding seed energy (EIS), probability availability of UTR (PAU), RBS coverage of length 11 (RBS11), and paired termini (PT)). The gray area depicts the 95 % confidence interval of the linear regression between the relative protein expression and each normalized feature value. Error bars represent standard deviation (n=3).

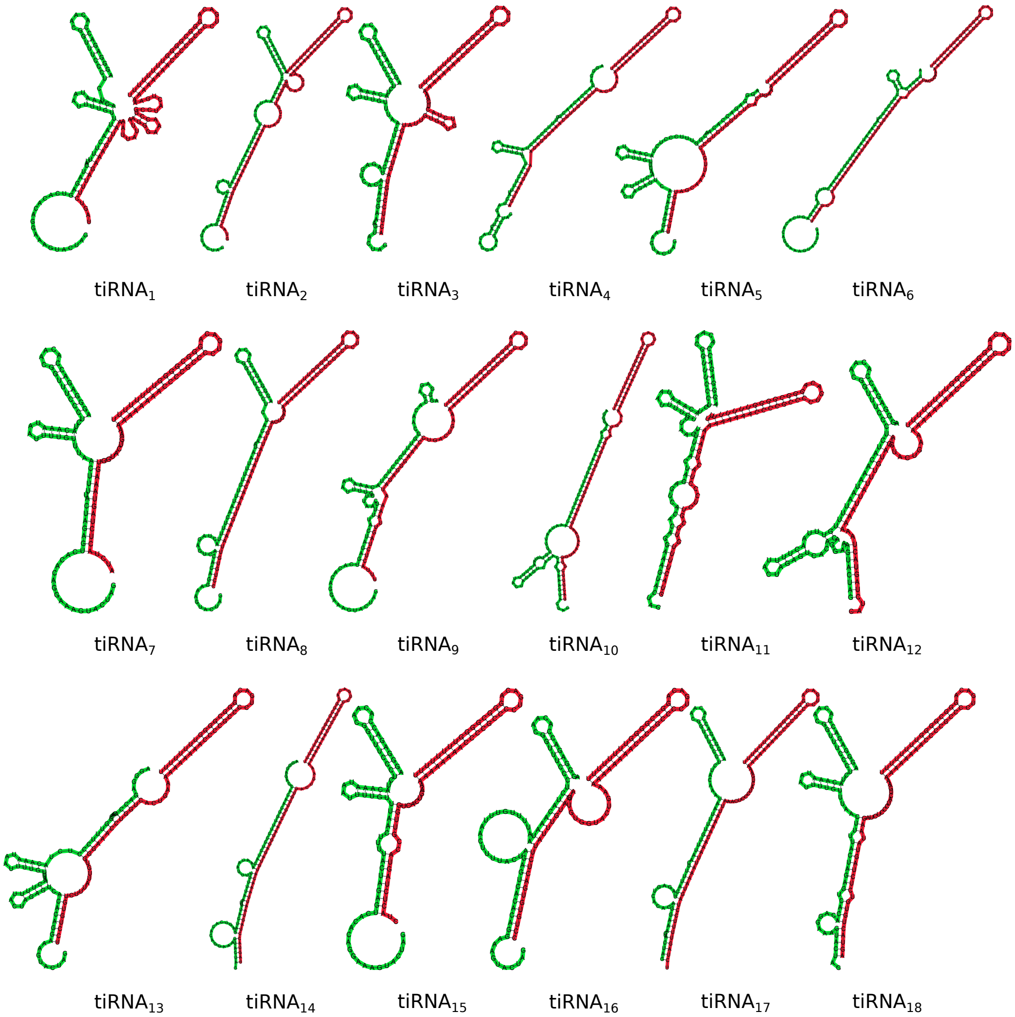

Fig G. Plot of the predicted minimum free energy (MFE) secondary structures of the 18 translation inhibiting RNAs (tiRNAs):UTR1 complexes. The UTR and tiRNA are depicted in green and red, respectively.

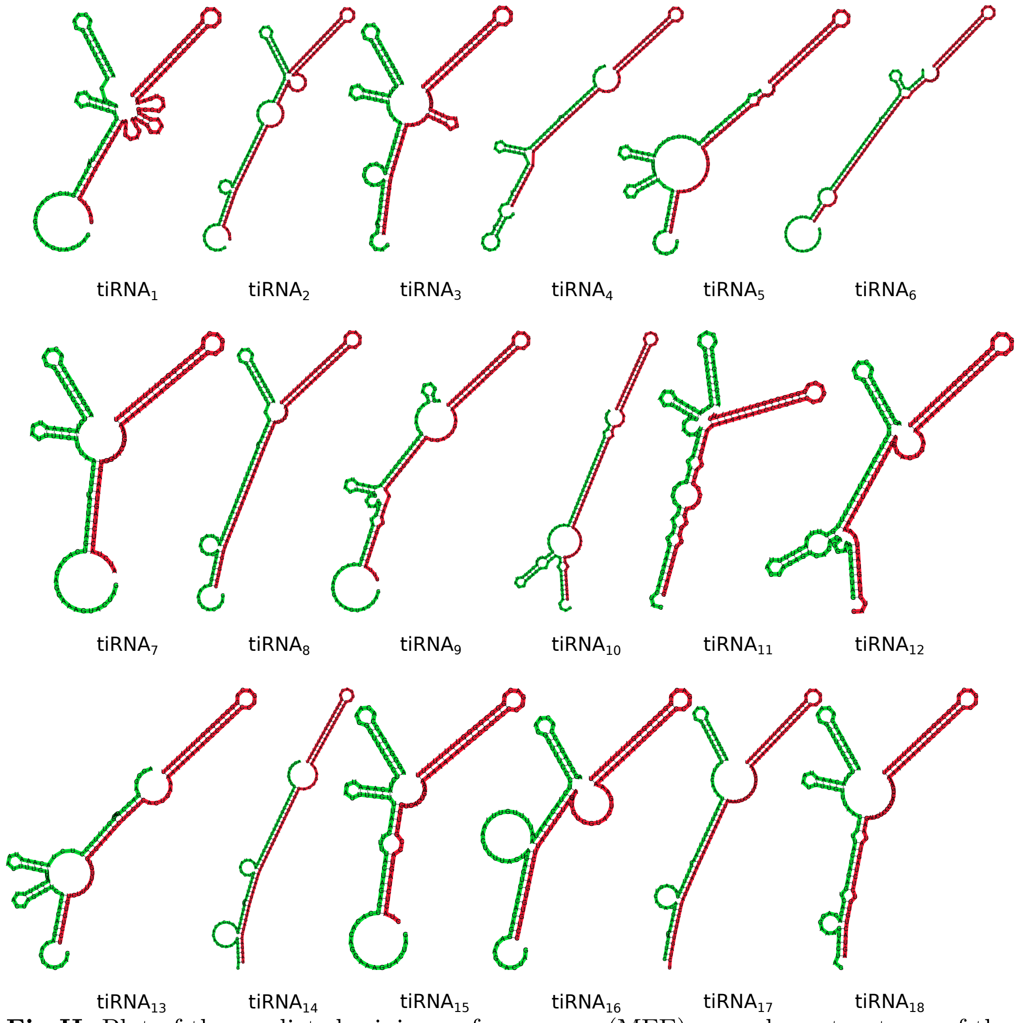

Fig H. Plot of the predicted minimum free energy (MFE) secondary structures of the 18 translation inhibiting RNAs (tiRNAs):UTR2 complexes. The UTR and tiRNA are depicted in green and red, respectively.

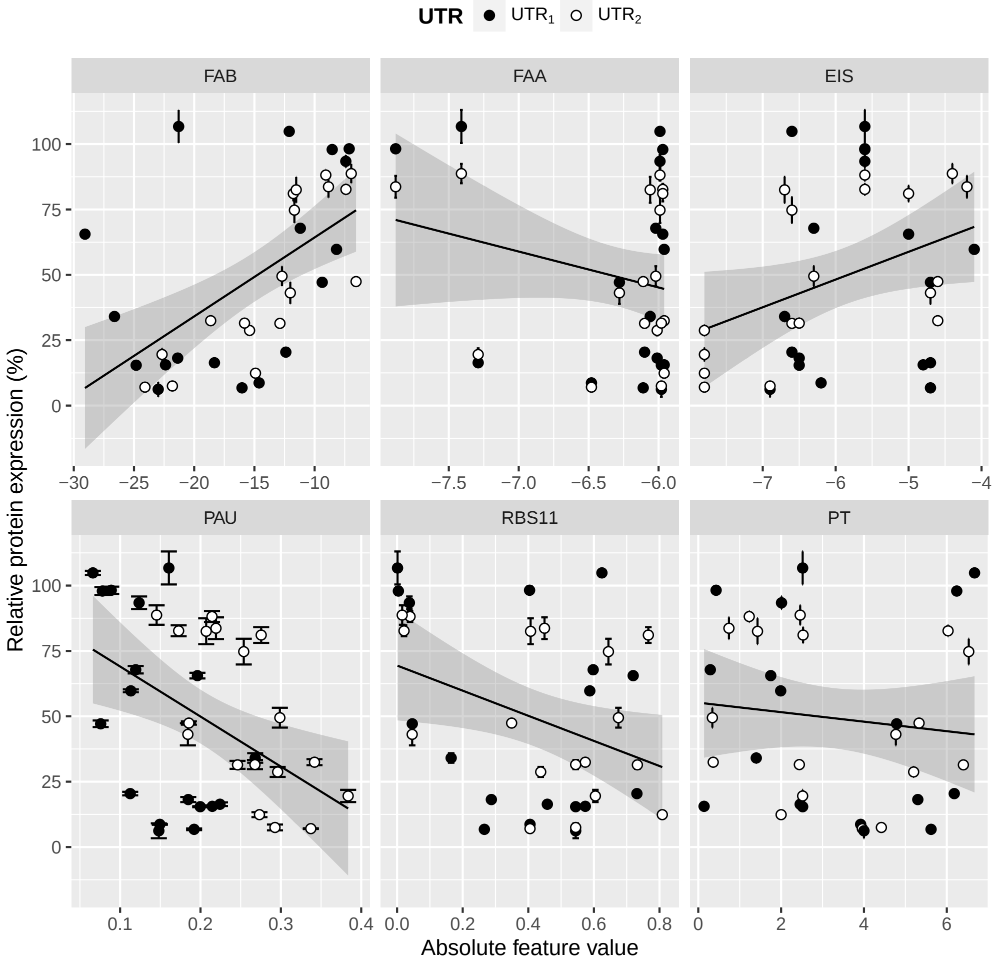

Fig I. Plot of the percentage relative expression of all data points against the absolute translation inhibiting RNA (tiRNA) features of the reduced feature set. The six factors used in the design of experiments (DOE) are the features in the reduced feature set (formation energy of the tiRNA-tiRNA dimer (FAA), formation energy of the tiRNA-UTR dimer (FAB), intermolecular binding seed energy (EIS), probability availability of UTR (PAU), RBS coverage of length 11 (RBS11), and paired termini (PT)). The gray area depicts the 95 % confidence interval of the linear regression between the relative protein expression and each absolute feature value. Error bars represent standard deviation (n=3).

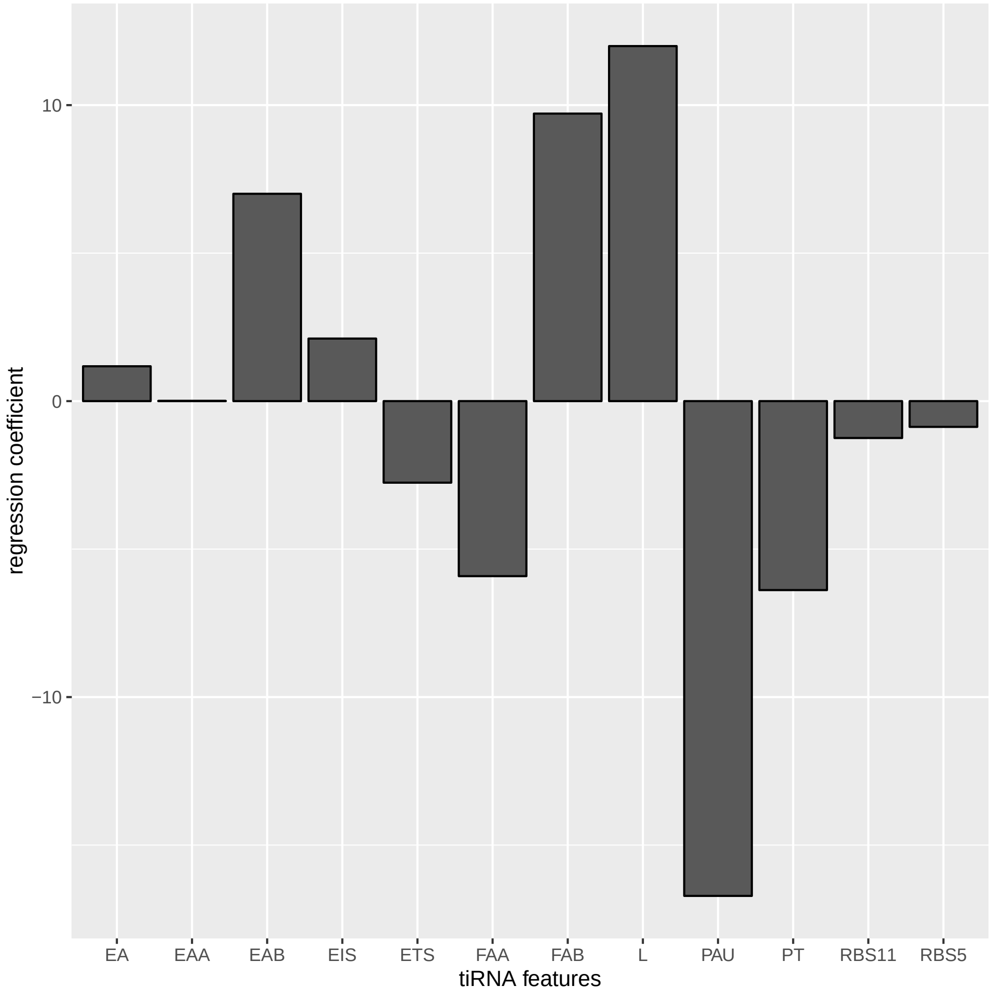

Fig J. The estimated partial least squares (PLS) regression coefficients of the eleven identified translation inhibiting RNA (tiRNA) features. Detailed definitions of all features (free energy of the tiRNA monomer (EA), free energy of the tiRNA-tiRNA dimer (EAA), free energy of the tiRNA-UTR dimer (EAB), formation energy of the tiRNA-tiRNA dimer (FAA), formation energy of the tiRNA-UTR dimer (FAB), total seed energy (ETS), intermolecular binding seed energy (EIS), probability availability of UTR (PAU), RBS coverage of length 5 (RBS5), RBS coverage of length 11 (RBS11), paired termini (PT), and tiRNA length (L)) are available in Table 1.

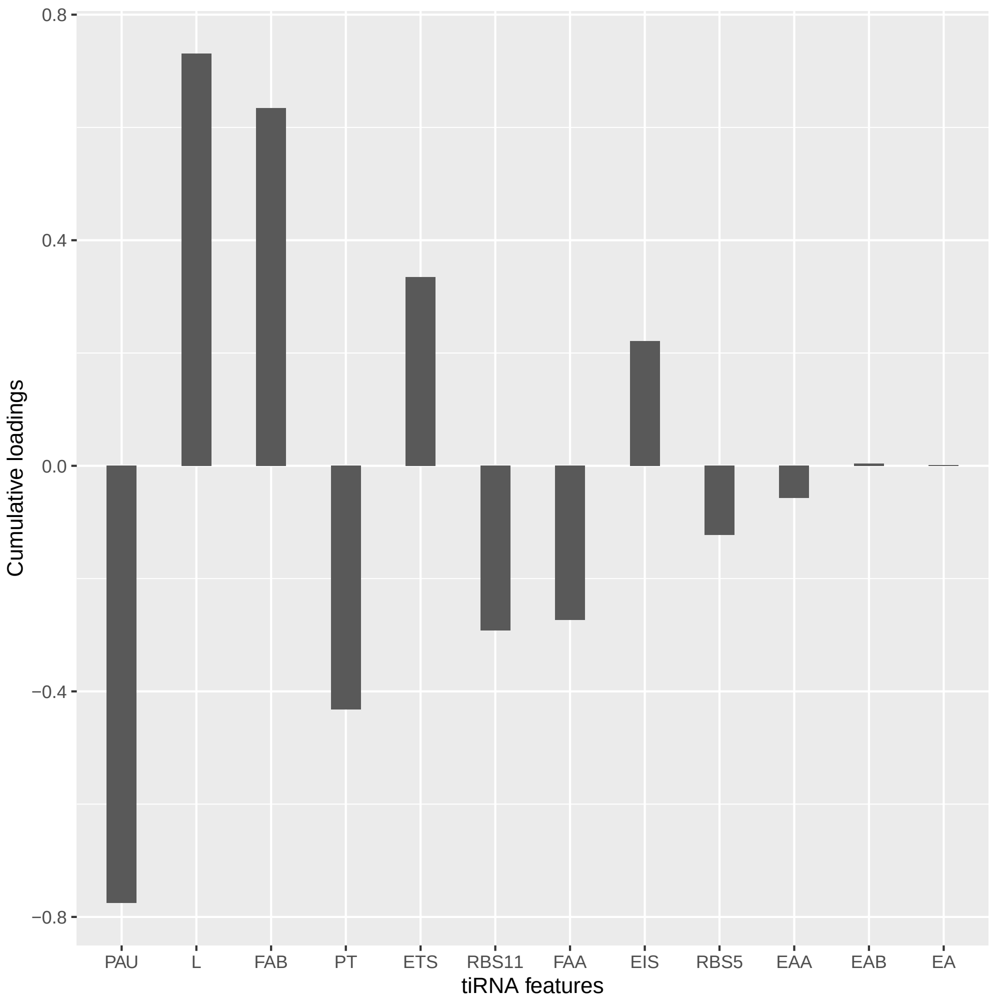

Fig K. Cumulative loadings of the four components used in the PLS model. Detailed definitions of all features (EA, EAA, EAB, FAA, FAB, ETS, EIS, PAU, RBS5, RBS11, PT, and tiRNA length (L)) are available in Table 1.

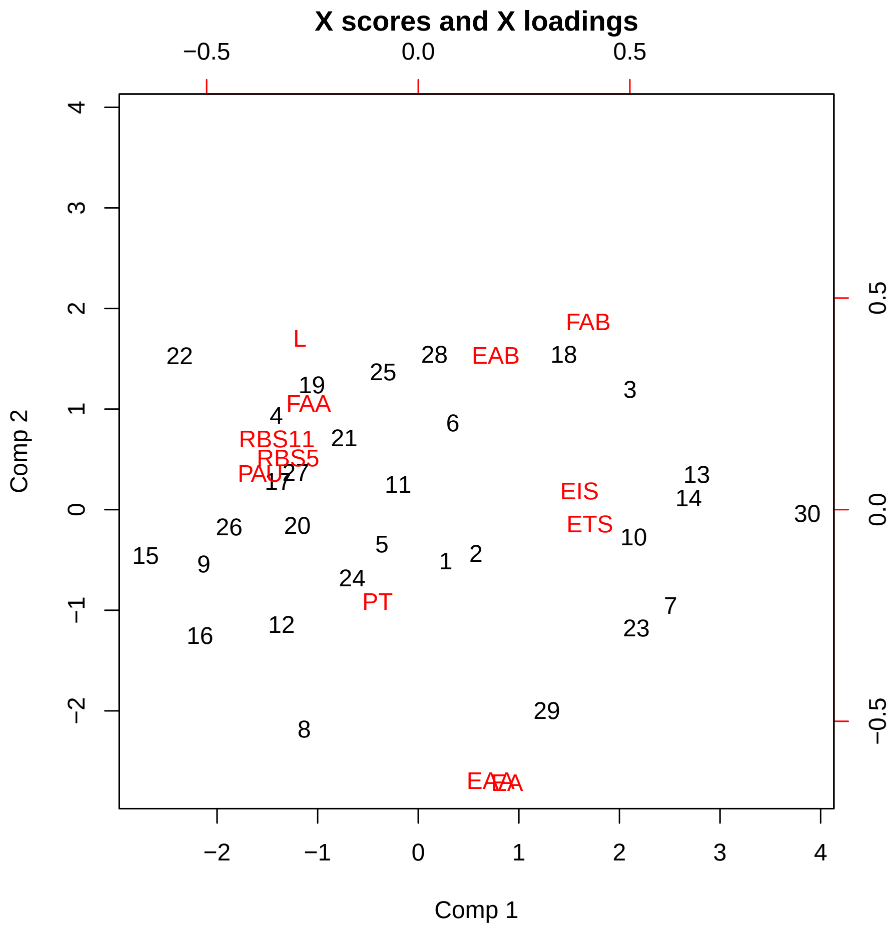

Fig L. Biplot of the first two components of the PLS regression model. Detailed definitions of all features (EA, EAA, EAB, FAA, FAB, ETS, EIS, PAU, RBS5, RBS11, PT, and tiRNA length (L)) are available in Table 1.

3 Supplementary Tables

Table A. Overview of all translation inhibiting RNA (tiRNA) molecules, comprising the experimental design to discover design rules for gene silencing using solely RNA.

**Name**	**RNA sequence (5'→3')**
$tiRNA_1$	UAGUCUUUAGAAAGUUAAAAUUAUUAAGGGAAACCUGCCU
$tiRNA_2$	GUACCUGUGUGGUAAAAGUUAAACAAUAUCCGUUGUGGUC
$tiRNA_3$	ACUUCCUGUAGUAAAAGUAUUGUGGUGUCU
$tiRNA_4$	AGUAAAGUUAAGAGGGAAACGUUGUGGUC
$tiRNA_5$	UUCUGUGUAGCAAAUUGUCCGUGUGGUCUC
$tiRNA_6$	UCUAGCCUGUUAAACAAAAUUAUUUGUGAGGGAAACUCCC
$tiRNA_7$	AUCUACUCUAGUAAAGUGGU
$tiRNA_8$	CCUGUGUUAAAAGUUAAACAAAAUUAUUGU
$tiRNA_9$	UUUACUCUAGUUAAAAUAGAGGGAAACCUG
$tiRNA_{10}$	UACUUUCGUGGUAAAAUAAUGUAGAGGGAAACCGUUGUGG
$tiRNA_{11}$	GUACUUUUGUGCGUCUCCCU
$tiRNA_{12}$	CAUCUAGUACUAAAAAAUUAUUUGUAGAGU
$tiRNA_{13}$	UCCUGUGUCUUUAUUGUGGGAAACCUGGUC
$tiRNA_{14}$	UAGUACCUAGUAAUUAAACAUGUAGAGGGAAACCGUUGUG
$tiRNA_{15}$	CUACUCUAGUACCGUUGGUC
$tiRNA_{16}$	CCUGUGUGACUCUUAUUUGUAGAUUGUGGU
$tiRNA_{17}$	UCUAGUUUUCCUAGUAAAAUUAAACAAAAUUAAACUCCCU
$tiRNA_{18}$	AGUACUGUGUCUCUAGUAUAAGAUCUCCCU

Table B. Overview of all plasmids used in this study.

Name	Content (5'→3')	Backbone
$pSilence_1$	proD - $tiRNA_1$ - BBa_B1006	pBR322
$pSilence_2$	proD - $tiRNA_2$ - BBa_B1006	pBR322
$pSilence_3$	proD - $tiRNA_3$ - BBa_B1006	pBR322
$pSilence_4$	proD - $tiRNA_4$ - BBa_B1006	pBR322
$pSilence_5$	proD - $tiRNA_5$ - BBa_B1006	pBR322
$pSilence_6$	proD - $tiRNA_6$ - BBa_B1006	pBR322
$pSilence_7$	proD - $tiRNA_7$ - BBa_B1006	pBR322
$pSilence_8$	proD - $tiRNA_8$ - BBa_B1006	pBR322
$pSilence_9$	proD - $tiRNA_9$ - BBa_B1006	pBR322
$pSilence_{10}$	proD - $tiRNA_{10}$ - BBa_B1006	pBR322
$pSilence_{11}$	proD - $tiRNA_{11}$ - BBa_B1006	pBR322
$pSilence_{12}$	proD - $tiRNA_{12}$ - BBa_B1006	pBR322
$pSilence_{13}$	proD - $tiRNA_{13}$ - BBa_B1006	pBR322
$pSilence_{14}$	proD - $tiRNA_{14}$ - BBa_B1006	pBR322
$pSilence_{15}$	proD - $tiRNA_{15}$ - BBa_B1006	pBR322
$pSilence_{16}$	proD - $tiRNA_{16}$ - BBa_B1006	pBR322
$pSilence_{17}$	proD - $tiRNA_{17}$ - BBa_B1006	pBR322
$pSilence_{18}$	proD - $tiRNA_{18}$ - BBa_B1006	pBR322
$pTarget_1$	proB - UTR_1 - *mKate2* - rnpB T1	pSC101
$pTarget_2$	proB - UTR_2 - *mKate2* - rnpB T1	pSC101
$pBlank_1$	/	pBR322
$pBlank_2$	*mKate2* - rnpB T1	pSC101

Table C. Overview of all important DNA sequences

Name	Sequence (5'→3')	Reference
mKate2	ATGGTTAGCGAGCTGATCAAAGAAAACATGCACATGAAAGCTGA<br>TCAAAGAAAACATGCACATGAAACTGTATATGGAAGGCACCGTG<br>AATAACCACCACTTTAAATGTACCAGCGAAGGTGAAAGCTGATC<br>AAAGAAAACATGCACATGAAACTGTATATGGAAGGCACCGTGAA<br>TAACCACCACTTTAAATGTACCAGCGAAGGTGAAGGTAAACCGT<br>ATGAAGGCACCCAGACCATGCGTATTAAAGCAGTTGAAGGTGGT<br>CCGCTGCCGTTTGCATTTGATATTCTGGCAACCAGCTTTATGTA<br>TGGCAGCAAAACCTTTATTAACCATACCCAGGGTATCCCGGATT<br>TTTTCAAACAGAGCTTTCCGGAAGGTTTTACCTGGGAACGTGTT<br>ACCACCTATGAAGATGGTGGTGTTCTGACCGCAACCCAGGATAC<br>CAGTCTGCAGGATGGTTGTCTGATTTATAATGTGAAAATTCGCG<br>GTGTGAACTTTCCGAGCAATGGTCCGGTTATGCAGAAAAAAACC<br>CTGGGTTGGGAAGCAAGCACCGAAACCCTGTATCCGGCAGATGG<br>TGGTCTGGAAGGTCGTGCAGATATGGCACTGAAACTGGTTGGTG<br>GTGGTCATCTGATTTGCAATCTGAAAACCACCTATCGTAGCAAA<br>AAACCGGCAAAAAATCTGAAAATGCCTGGCGTGTATTATGTTGA<br>TCGTCGTCTGGAACGTATTAAAGAGGCAGATAAAGAAACCTATG<br>TGGAACAGCATGAAGTTGCAGTTGCACGTTATTGTGATCTGCCG<br>AGCAAACTGGGTCACCGCTGATAA	[12]
proB	CTAGAGCACAGCTAACACCACGTCGTCCCTATCTGCTGCCCTAG<br>GTCTATGAGTGGTTGCTGGATAACTTTACGGGCATGCATAAGGC<br>TCGTAATATATATTC	[10]
proD	CTAGAGCACAGCTAACACCACGTCGTCCCTATCTGCTGCCCTAG<br>GTCTATGAGTGGTTGCTGGATAACTTTACGGGCATGCATAAGGC<br>TCGTATAATATATTC	[10]
rnpB T1	TCGGTCAGTTTCACCTGATTTACGTAAAAACCCGCTTCGGCGGG<br>TTTTTGCTTTTGGAGGGGCAGAAAGATGAATGACTGTC	[11]
BBa_B1006	AAAAAAAAACCCCGCCCCTGACAGGGCGGGGTTTTTTTT	[11]
UTR_1	AGGGAGACCACAACGGTTTCCCTCTACAAATAATTTTGTTTAAC<br>TTTTACTAGAGTCACACAGGAAAGTACTAG	[10]
UTR_2	AGGGAGATTGACTTTTACTAGAGTCACACAGGAAAGTACTAG	this work

Table D. Detailed description of the $2^{6\text{-}2}$ fractional factorial design to unravel translation inhibiting RNA (tiRNA) design principles. This experimental design for six 2-level factors comprises 16 regular runs ($tiRNA_{1\text{-}16}$) and two center points ($tiRNA_{17}$ and $tiRNA_{18}$). The six factors used in the design of experiments (DOE) are the features in the reduced feature set (formation energy of the tiRNA-tiRNA dimer (FAA), formation energy of the tiRNA-UTR dimer (FAB), intermolecular binding seed energy (EIS), probability availability of UTR (PAU), RBS coverage of length 11 (RBS11), and paired termini (PT)).

	FAB	FAA	EIS	PAU	RBS11	PT
$tiRNA_1$	-1	-1	-1	-1	-1	-1
$tiRNA_2$	1	-1	-1	-1	1	1
$tiRNA_3$	-1	1	-1	-1	1	1
$tiRNA_4$	1	1	-1	-1	-1	-1
$tiRNA_5$	-1	-1	1	-1	1	-1
$tiRNA_6$	1	-1	1	-1	-1	1
$tiRNA_7$	-1	1	1	-1	-1	1
$tiRNA_8$	1	1	1	-1	1	-1
$tiRNA_9$	-1	-1	-1	1	-1	1
$tiRNA_{10}$	1	-1	-1	1	1	-1
$tiRNA_{11}$	-1	1	-1	1	1	-1
$tiRNA_{12}$	1	1	-1	1	-1	1
$tiRNA_{13}$	-1	-1	1	1	1	1
$tiRNA_{14}$	1	-1	1	1	-1	-1
$tiRNA_{15}$	-1	1	1	1	-1	-1
$tiRNA_{16}$	1	1	1	1	1	1
$tiRNA_{17}$	0	0	0	0	0	0
$tiRNA_{18}$	0	0	0	0	0	0

Table E. Untranslated regions (UTRs) used in this study

Name	Sequence (5'→3')
UTR_1	AGGGAGACCACAACGGUUUCCCUCUACAAAUAAUUUUGUUUAACUUU UACUAGAGUCACACAGGAAAGUACUAG
UTR_2	AGGGAGAUUGACUUUUACTAGAGTCACACAGGAAAGUACUAG

Table F. Coefficients and scaling factors of the partial least squares (PLS) model constructed in this study.

Feature	Coefficient	Scale
intercept	80.8268	
EA	1.1770	1.5677
EAB	7.0045	11.6650
EAA	0.0069	2.9119
FAB	9.7145	6.2888
FAA	-5.9140	0.6206
ETS	-2.7558	1.1525
EIS	2.1132	1.1253
PAU	-16.7172	0.0825
RBS5	-0.8708	0.3216
RBS11	-1.2471	0.2534
PT	-6.3849	2.0418
L	11.9954	7.6336

References

1. Wuchty S, Fontana W, Hofacker IL, Schuster P, et al. Complete suboptimal folding of RNA and the stability of secondary structures. Biopolymers. 1999;49(2):145–165.

2. Mückstein U, Tafer H, Hackermüller J, Bernhart SH, Stadler PF, Hofacker IL. Thermodynamics of RNA–RNA binding. Bioinformatics. 2006;22(10):1177–1182.

3. Johnson E, Srivastava R. Volatility in mRNA secondary structure as a design principle for antisense. Nucleic Acids Research. 2013;41(3):e43–e43.

4. Giegerich R, Voß B, Rehmsmeier M. Abstract shapes of RNA. Nucleic acids research. 2004;32(16):4843–4851.

5. Huthoff H, Berkhout B. Two alternating structures of the HIV-1 leader RNA. RNA. 2001;7(01):143–157.

6. Evers D, Giegerich R. RNA movies: visualizing RNA secondary structure spaces. Bioinformatics. 1999;15(1):32–37.

7. Hofacker IL, Fontana W, Stadler PF, Bonhoeffer LS, Tacker M, Schuster P. Fast folding and comparison of RNA secondary structures. Monatshefte für Chemie/Chemical Monthly. 1994;125(2):167–188.

8. Nakashima N, Tamura T. Conditional gene silencing of multiple genes with antisense RNAs and generation of a mutator strain of *Escherichia coli*. Nucleic Acids Research. 2009;37(15):e103–e103.

9. Nakashima N, Tamura T, Good L. Paired termini stabilize antisense RNAs and enhance conditional gene silencing in Escherichia coli. Nucleic acids research. 2006;34(20):e138–e138.

10. Davis J, Rubin A, Sauer R. Design, construction and characterization of a set of insulated bacterial promoters. Nucleic acids research. 2011;39(3):1131–1141.

11. Cambray G, Guimaraes JC, Mutalik VK, Lam C, Mai QA, Thimmaiah T, et al. Measurement and modeling of intrinsic transcription terminators. Nucleic Acids Research. 2013;41(9):5139–5148.

12. Shcherbo D, Murphy C, Ermakova G, Solovieva E, Chepurnykh T, Shcheglov A, et al. Far-red fluorescent tags for protein imaging in living tissues. Biochem J. 2009;418:567–574.

13. Registry of Standard Biological Parts; 2016. `http://parts.igem.org/`.
